# Multiobjective Resource-Constrained Project Scheduling with a Time-Varying Number of Tasks

**DOI:** 10.1155/2014/420101

**Published:** 2014-04-15

**Authors:** Manuel Blanco Abello, Zbigniew Michalewicz

**Affiliations:** ^1^School of Computer Science, University of Adelaide, Adelaide, SA 5000, Australia; ^2^Institute of Computer Science, Polish Academy of Sciences, 5 Jana Kazimierza, 01-248 Warsaw, Poland; ^3^Polish-Japanese Institute of Information Technology, Street Koszykowa 86, 02-008 Warsaw, Poland

## Abstract

In resource-constrained project scheduling (RCPS) problems, ongoing tasks are restricted to utilizing a fixed number of resources. This paper investigates a dynamic version of the RCPS problem where the number of tasks varies in time. Our previous work investigated a technique called mapping of task IDs for centroid-based approach with random immigrants (McBAR) that was used to solve the dynamic problem. However, the solution-searching ability of McBAR was investigated over only a few instances of the dynamic problem. As a consequence, only a small number of characteristics of McBAR, under the dynamics of the RCPS problem, were found. Further, only a few techniques were compared to McBAR with respect to its solution-searching ability for solving the dynamic problem. In this paper, (a) the significance of the subalgorithms of McBAR is investigated by comparing McBAR to several other techniques; and (b) the scope of investigation in the previous work is extended. In particular, McBAR is compared to a technique called, Estimation Distribution Algorithm (EDA). As with McBAR, EDA is applied to solve the dynamic problem, an application that is unique in the literature.

## 1. Introduction

In executing an air traffic schedule, bad weather or emergencies might occur whereby to-be-executed activities in this schedule are no longer feasible. Many real-world problems are set in this type of dynamic scenario where their objectives, constraints, or even dimensions may change in time [1–3]. This is particularly true for some* resource-constrained project scheduling* (RCPS) problems, a class of problems that have ongoing tasks restricted to utilizing a limited number of resources [4]. Some RCPS problems are NP-hard which are often approached using modern heuristic methods, in particular,* Evolutionary Algorithms* (EA) [5].

Many researches [6–9] have investigated scheduling problems that have fixed dimensions. However, there are important scheduling problems and each of them has varying dimensions. For example, suppose that in implementing a resource-constrained schedule for an edifice construction, a large buried object is found. As a consequence, a new task to remove this object must be performed before other tasks in the schedule can continue or commence. If each variable in the problem of determining a schedule corresponds to a task, then the revision of the schedule to accommodate a new task entails a new problem that may have a greater number of variables (dimensions) than the original problem.

Many scheduling problems are multiobjective [10–13]. For example, in creating the edifice construction schedule above, one objective is to reduce the construction duration which could be accomplished by employing large number of laborers. However, due to overhead expenses (e.g., hazard fee) per laborer, the construction cost for many laborers could be higher than that for a few laborers for the same total man-hours. Now, if another objective is to reduce the construction cost which could be accomplished by hiring fewer laborers, the construction duration could increase. Thus, in this example, the two conflicting objectives (minimization of construction cost and duration) cannot possibly be achieved simultaneously. A problem that requires simultaneous optimization of conflicting objectives is referred to as* multiobjective optimization* (MOO) problem [1].

Let Φ^2^ denote the biobjective dynamic RCPS problem where minimization of the schedule cost and duration are the conflicting objectives and where the number of tasks varies in time, a variation that brings about a change in the dimension of this problem. Our previous work [14] investigated the performance (solution-searching ability) of the memory and EA-based technique called* mapping of task IDs for centroid-based adaptation with random immigrants* (McBAR) in solving the Φ^2^ problem. However, this investigation was performed on few instances of Φ^2^. Consequently, only a few characteristics of McBAR were found under the dynamics of this problem. Further, only a few techniques were being compared to McBAR with respect to its performance in solving the problem.

The major goals of this paper are to

1. legitimize some subalgorithms constituting McBAR. Legitimization of a subalgorithm of a technique is to manifest the decline in the effectiveness of this technique in solving a problem when the subalgorithm is replaced;
2. extend the investigations in our previous work [14]. In particular, add the techniques being compared to McBAR with the technique that utilizes* Estimation Distribution Algorithm* (EDA) [15]. As with McBAR, this additional technique is applied to solve the Φ^2^ problem. This application is unique in the literature.

This paper is organized as follows.  Section 2 explores the knowledge useful to understand the problems and methods presented in this paper.  Section 3 provides information on the problems and the methods used to solve these problems. The results obtained for applying these methods are described and investigated in Section 4, together with the demonstration of the way in which the goals above are accomplished.  Section 5 presents the conclusion of this work.

## 2. Background Knowledge

The following five subsections contain general and specific background knowledge helpful for understanding the techniques and problems discussed in this paper.  Section 2.1 investigates some RCPS problems, each with a time-varying number of tasks, and gives special emphasis to approaches applied to solve these problems.  Section 2.2 presents the memory-based approach, upon which McBAR is founded.  Section 2.3 provides information on some multiobjective optimization problems which are related to that of Φ^2^. EDA is presented in Section 2.5 including its application to some RCPS problems.

### 2.1. Resource-Constrained Project Scheduling (RCPS)

**Figure 1:**
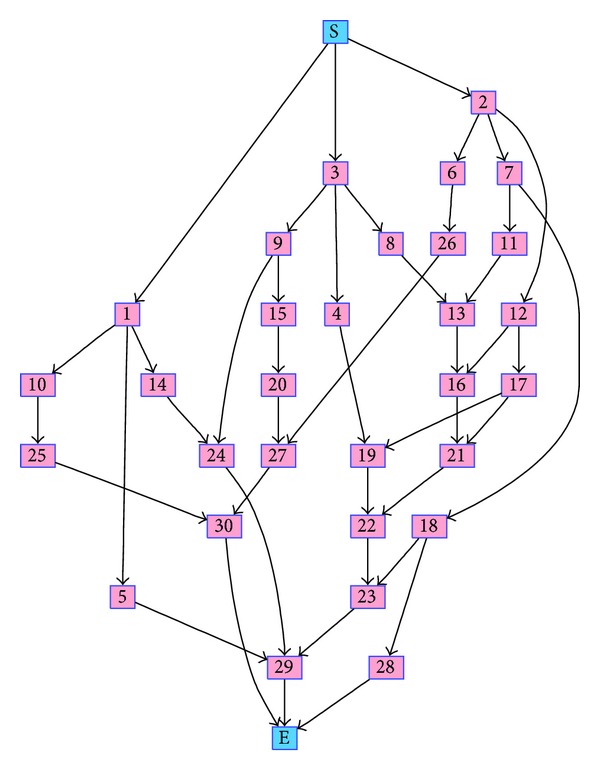
Original task precedence network.

Scheduling as a solution to any RCPS problem is composed of tasks obeying some precedence relationship, such as that exemplified in Figure 1. In this figure, boxed numbers are IDs of tasks of an RCPS; directed links signify precedence relationships of these tasks; and labels “S” and “E” correspond to starting and ending tasks, respectively. Note that any task precedence network that will be mentioned from here onwards is of the form just described. RCPS tasks are characterized by several attributes such as, duration, starting time, and required resources, for example, personnel, materials, and fuel. In RCPS, the number of resources of the same type utilized by all ongoing tasks are constrained not to exceed a predefined limit.

A schedule implemented in a dynamic environment can turn into an infeasible or a low quality one, for example, one involving a high cost of implementation. Thus, there could be a need to revise this schedule. However, the following rule must be taken into account in the revision.

 Schedule revision must judiciously be made to avoid the high cost of altering its in-use components which thus may be preserved [16].

Given an RCPS problem, let the total number of tasks be defined as the sum of the number of finished, ongoing, and to-be-executed tasks at the moment of change in the dynamic environment that sets this problem. Note that task cancellation is not considered in this paper. In our previous work [14, 17] as well as in this paper, we consider three entities that could change in the environment: resource availability, task duration, and total number of tasks. The change in the total number of tasks is given the major emphasis.

#### 2.1.1. Time-Varying Task Number

There had been several reactive scheduling approaches to revise schedules to cope with the effects of the time-varying number of tasks. For example, in the job-shop scheduling problem in [18], during the EA process of evolving a population of schedules, genes which correspond to new/old jobs that arrived/finished are inserted/removed to/from genotypes which correspond to the schedules. The resulting population is then evolved further to search for new high-quality schedules. It is worth noting that despite the genotype alteration, significant improvement on the EA's search convergence and solutions/schedules quality was found [16, 19–21]. In [22], tasks in processors of a distributed computing system arrive randomly and are put on queue. These tasks are then processed by batch that has varying number of tasks. This process follows a schedule created through Genetic Algorithm to obtain minimal combined execution time of tasks in every batch. In [23], genes that correspond to new tasks are also inserted into genotypes which are then processed by EA to obtain a new high-quality schedule for multiresource scheduling with cumulative constraints. In this EA process, lateness of the new schedule is minimized and at the same time, important properties of the original schedule are preserved. EDA was applied to solve a non-RCPS problem whose dimension changes in time [24, 25]. However, the objective functions utilized in this application were simple. More techniques in revising schedules, to cope with the variation of the number of tasks in the environments that set these schedules, can be found in [4].

#### 2.1.2. Schedule Generation

We now discuss a process of generating schedules referred to as* serial schedule generation scheme* (SSGS). SSGS is also a popular method to form initial populations used to solve RCPS problems through EA [26]. Before proceeding, some terms will be defined. Let a genotype be viewed as an ordered set of slots to which IDs, of tasks that comprise a schedule, will be filled in consecutively (e.g., from leftmost slot to the right). Consider a genotype devoid of IDs. Once an ID is filled in, its corresponding task will be called a scheduled task. A root task is defined as the task from which all other tasks succeed, for instance, the task labeled “S” in Figure 1. The root task is considered as a scheduled task even though its ID is not filled in to the genotype. Eligible tasks have IDs not yet filled in to the genotype and are the immediate successors of scheduled tasks and/or the root task. For example, given the task precedence in Figure 1, if scheduled tasks have IDs 1, 2 and “S,” the set of eligible tasks have IDs in set *E*_*t*_ = {5,10,14} ∪ {6,7, 12} ∪ {3} whose subsets (indicated by { }) contain ID/s of task/s. These tasks are the immediate successors to their corresponding (based on the figure) scheduled and root tasks.

A version of the SSGS algorithm to create a resource-constrained schedule is depicted in Algorithm 1. It starts by determining the set *E*_*t*_ of IDs of eligible tasks not yet with any scheduled task, except the root task. This implies that, at the start, *E*_*t*_ is composed of IDs of tasks immediately succeeding the root task. In the last example, the set of eligible tasks at this stage is *E*_*t*_ = {1,2, 3}. Let *N* be the number of tasks to be scheduled. A loop is then executed *N* times to fill all slots of the genotype utilized in this algorithm. In each cycle, indexed *g* of this loop a task ID *j* is selected randomly from *E*_*t*_ and placed to the genotype at slot indexed *g*. Then set the starting time st_*j*_ of the task that corresponds to this ID to be the earliest time *t*′ later than the maximum end time—start time st_*i*_ plus duration *d*_*i*_^*r*^—of all its predecessors whose IDs are in the set Pred(*j*). Further, the starting time st_*j*_ is such that there are enough available resources, for task with ID *j*, to utilize over the entire execution period of this task. Note that some resources an RCPS environment could already been allocated to some scheduled tasks and hence could be unavailable. Task with ID *j* is now considered scheduled. And the last step in the loop is to update *E*_*t*_. After *N* cycles, the genotype is completely filled and the starting times of tasks that correspond to IDs in the genotype are determined. Thus, a schedule is formed.

### 2.2. Memory-Based Approaches

EA techniques which utilize memory record relevant information that corresponds to previous problems they had solved or to past evolutionary generations they had gone through. This information is retrieved to assist in solving a current problem or a problem at a current evolutionary generation [27]. Suppose that a previous planning problem *P*_prev_ and a current problem *P*_curr_ are close, based on a certain measure. It could then be expected that the fitness landscapes that correspond to these problems are also close, by a different measure. Thus, the solutions to these problems could also be close, based on another measure. By this proximity, few EA cycles could be required to evolve an initial population, containing solutions or representative of solutions to *P*_prev_, to become solutions to *P*_curr_ [6, 28]. This expectation underlies several memory-based approaches in EA [6, 28–30].

Performances (solution-searching abilities) of memory-based EA approaches are highly dependent on the diversity of population which they create and then evolve [31]. They can be undertaken either in explicit or implicit styles.

#### 2.2.1. Explicit Memory-Based Approach

Explicit memory-based Approach defines how information produced by EA in solving problems and/or information about environments that set these problems is stored and retrieved [32]. The combination of competitive learning [33] and explicit memory-based EA approach was applied in [29, 32] to solve some problems in an artificial dynamic environment. An explicit memory-based EA approach in [34] is based from the human immune system. For each previous form of a dynamic knapsack problem that has analogues of antigens (molecules foreign to the immune system), a representative of the analogues of the pathogens in the system is kept in a memory and is used for solving a current form of the problem. The basic approach in [35] was to search for and then store to memory solutions suitable for many considered environmental states. Based on some mathematical measures, one of the solutions measured as being the most suitable was retrieved from the memory and then implemented on a current environmental state. However, if none of these solutions was measured as being suitable, a new optimal solution was searched. In [18], a prioritized list of categories of scheduling task properties was created and stored in a memory. Tasks to be executed had their properties matched to the categories to produce a prioritized list of tasks from which an initial population was formed and then evolved to determine the schedule of the tasks. The system in [28] continuously learns of changes in an environment to dynamically update its knowledge base of pairs of environmental properties and solutions to problem set in this environment. A pair that contained environmental properties that match, based on a mathematical measure, those of the current environmental state was retrieved from the knowledge base. The solutions contained in the retrieved pairs were then utilized to form an initial population which was evolved through EA to search for optimal solutions to a problem set in the current state of the environment. A system in [30], closely related to that in [28], was applied to some dynamic resource allocation problem in a command and control environment. This allocation problem considered risk and cost in a project implementation, factors which were lumped into one objective function.

#### 2.2.2. Implicit Memory-Based Approach

Implicit memory-based approach defines the way in which representatives of information produced by EA for solving problems are stored and retrieved [32]. As in explicit style, this information may or may not be added to the information on the environment that sets the problems. In the scheduling domain, implicit style has advantage over explicit style because, in a dynamic scenario, the schedule produced by explicit style will swiftly become irrelevant due to variation in the priority and precedence order of schedule components [18].

Multistranded chromosome (polyploidy) was utilized in [36–38] to store representatives of information that correspond to past environmental changes. This utilization was shown to be useful in searching solutions, through Genetic Algorithm, to some problems in a dynamic environment. Chromosomes utilized in [39] have a multilevel genetic structure which serve as long-term memory and facilitate quick adaptation of a function optimizer to environmental changes.

### 2.3. Multiobjective Optimization (MOO)

Let us now explore some important features of the MOO problem. Let **x** = {*x*_1_, *x*_2_,…, *x*_*M*_} ∈ *C* ^*M*^ be referred to as decision vector (e.g., genotype) of decision variables *x*_*i*_ (e.g., ID of task in a schedule) and *f*_*k*_(**x**) as the *k*th objective function which relates a decision vector to the *k*th objective value (e.g., cost to implement an entire schedule). The objective vector **f**(**x**) for a *K*-objective problem is denoted by

(1) $$\begin{array}{c} f\left(x\right)=\langle f_{1}\left(x\right),f_{2}\left(x\right),\ldots ,f_{k}\left(x\right),\ldots ,f_{K}\left(x\right)\rangle . \end{array}$$

The concept of dominance of a solution is relevant for comparing the quality of this solution to another solution of the same MOO problem. To explain this concept, consider two sets of indices, *C* = {*i*_1_, *i*_2_,…, *i*_*L*_} and *D* = {*j*_1_, *j*_2_,…, *j*_*E*_} where *L* + *E* = *K*, *C*∩*D* = *∅*, and *C* ∪ *D* = {1,2,…, *K*}. Let the indices be those of the objective functions *f*_*k*_ contained in the expression of **f**(**x**) in (1). A solution **x**_1_ dominates another **x**_2_, denoted by **x**_1_⪯**x**_2_, if ∃ a nonempty set *C* where *f*_*i*_(**x**_1_) < *f*_*i*_(**x**_2_)  ∀*i* ∈ *C* and *f*_*j*_(**x**_1_) = *f*_*j*_(**x**_2_)  ∀*j* ∈ *D*, that is, if there is one or more of the objective functions each yielding objective value at **x**_1_ less than that at **x**_2_ and if the rest of the objective functions each yielding objective value at **x**_1_ equaled that at **x**_2_. This definition is applicable when objectives are to be minimized. Otherwise, the inequality sign will be reversed and “greater than” will be used instead of “lesser than" in the definition of dominance.

For example, suppose that there are two conflicting objectives to minimize, schedule duration and cost, which correspond to objective functions *f*_1_(**x**) and *f*_2_(**x**), respectively, where **x** is a schedule. If the cost in implementing schedule **x**_1_ is lesser than that of schedule **x**_2_, that is, *f*_1_(**x**_1_) < *f*_1_(**x**_2_) and the duration in accomplishing schedule **x**_1_ is shorter than that of schedule **x**_2_, that is, *f*_2_(**x**_1_) < *f*_2_(**x**_2_), then **x**_1_⪯**x**_2_; that is, schedule **x**_1_ is of better quality than **x**_2_. In this example, the sets of indices *C* and *D* in the definition of dominance above are equal to {1,2} and *∅*, respectively.

If **x**_1_ does not dominate **x**_2_ and **x**_2_ also does not dominate **x**_1_, they are referred to as nondominated. A set of nondominated solutions is called* nondominated set* (NDS). There could be many solutions to a MOO problem. At least one of them may constitute an NDS [40]. In practice, guided by his/her experience and intuition, a decision maker may choose one solution from an NDS to implement in his/her field of interest. From here onwards, let the chosen solution be referred to as* chosen schedule* if it is in the context of scheduling.

One way to compare the quality of the set *A* of solutions, obtained by the method *T*_*A*_, to another set *B* of solutions, obtained by another method *T*_*B*_, is through the set coverage. Let *D*(*A*, *B*) be the set containing elements of *B* which are dominated by an element in *A*,

(2) $$\begin{array}{c} D\left(A,B\right)=\left\{b\in B\;|\;\exists \;a\in A:a⪯b\right\}. \end{array}$$

The set coverage is defined as

(3) $$\begin{array}{c} \text{SC}\left(A,B\right)=\frac{\left|D\left(A,B\right)\right|}{\left|B\right|}, \end{array}$$

where | | is the set cardinality. This definition can also be applied to monoobjective optimization.

Based on (3), the set coverage has a range of 0 ≤ SC(*A*, *B*) ≤ 1. It will be convenient for later discussions to have a set coverage-related quantity that has a range symmetric around zero. For this purpose, we define

(4) $$\begin{array}{c} \text{dSC}\left(A,B\right)=\text{SC}\left(A,B\right)-\text{SC}\left(B,A\right), \end{array}$$

where dSC(*A*, *B*) is referred to as differential set coverage which is antisymmetric on its arguments. Assuming that |*A*| = |*B*| and based on (4), dSC(*A*, *B*) has a range of values,

(5) $$\begin{array}{c} -1\leq \text{dSC}\left(A,B\right)\leq 1. \end{array}$$

We then take the following.

 Suppose that *A* and *B* are sets of solutions computed, respectively, by techniques *T*_*A*_ and *T*_*B*_ under similar conditions. Technique *T*_*A*_ performs better than technique *T*_*B*_ under these conditions if dSC(*A*, *B*) > 0.

### 2.4. Multiobjective Evolutionary Algorithm (MOEA)

As was pointed out in the Introduction, some RCPS problems which are NP-hard are often solved using EA. Further, recall also that Φ^2^ is an RCPS problem compounded by multiobjectivity. A class of EA-based methods suitable to solve multiobjective NP-hard problems is referred to as* Multiobjective Evolutionary Algorithm* (MOEA). A popular member of MOEA is the* Nondominated Sorting Genetic Algorithm-II* (NSGA-II) [41]. Being a type of EA, NSGA-II has an evolutionary process which starts on an initial population. Further, given an evolutionary stage, the selection process in NSGA-II gives preference to individuals (solutions) far from crowded individuals in the search space associated with the problem being solved through NSGA-II. This preference helps diversify offspring existing into the next evolutionary cycle [41].

**Figure 2:**
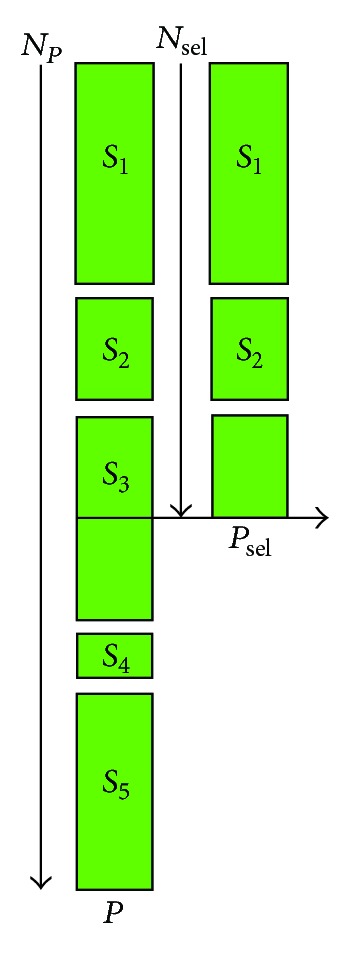
NSGA-II selection.

*Selection.* The selection mechanism of NSGA-II is described as follows: at each evolutionary cycle of NSGA-II, fast nondominated sorting is applied to population *P*, and as a consequence, each individual in *P* is endowed with integral Pareto rank *k* > 0 and crowding distance [41]. Let *S*_*k*_⊆*P* be a set of individuals each having a Pareto rank of *k*. Further, let *P*_sel_ be the population of selected individuals. Starting from *S*_1_, each *S*_*k*_ is included in ascending rank *k* to *P*_sel_ which starts from empty. This inclusion will stop before including *S*_*q*_ which will result in |*P*_sel_| > *N*_sel_, the number of individuals to be selected. If at the moment of stopping |*P*_sel_| < *N*_sel_, then individuals in *S*_*q*_ will be sorted in descending order of their crowding distances, thereby *S*_*q*_ becomes an ordered set *Srt*_*q*_. Then *Sfirst*, the set of first *N*_sel_ − |*P*_sel_| elements of *Srt*_*q*_, will be included in *P*_sel_. Thereby, *P*_sel_ have exactly *N*_sel_ elements. As exemplified in Figure 2, if *S*_3_ is included in *P*_sel_, |*P*_sel_| > *N*_sel_, such that only its subset is included to *P*_sel_. Now, if at the moment of stopping, *P*_sel_ has *N*_sel_ number of elements already, then there is no need to include any element of *S*_*q*_.

### 2.5. Estimation Distribution Algorithm (EDA)

*Estimation Distribution Algorithm* (EDA) is an evolutionary heuristic which, instead of using EA operators, makes use of sampling and estimation of* probability density functions* (PDFs) to create its next-generation population [15]. It is a heuristic that has the ability to detect and preserve good quality building blocks of chromosomes [42], an ability that could be important in some applications [15]. It has not been applied to solve the Φ^2^ problem. Its application to solve these problems is unique in the literature.

**Figure 3:**
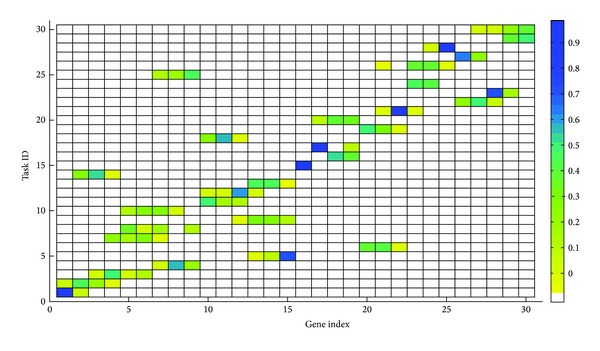
Sample task ID distribution.

Before discussing a particular algorithm of EDA, let us describe Figure 3. Suppose that genes in genotypes that correspond to individuals in a given population are task IDs. Each color block in this figure signifies the density of the genotypes that have task ID equal to the task ID in the vertical axis at gene index in the horizontal axis. Let the matrix of density depicted in the figure be referred to as the probability matrix.

Algorithm 2 presents a particular EDA algorithm. Beginning at its first generation *i* = 0, EDA creates a probability matrix Pm_*i*_ with equal entries. This probability matrix is then sampled to form a set of genotypes *G*_*i*_, sized *N*. Genotypes in *G*_*i*_ are then selected to form another set *G*sub_*i*_ of genotypes, sized ⌈*ρN*⌉, where ⌈·⌉ is a round-up operator and 0 < *ρ* ≤ 1 is a constant. Their selection is based on the fitness of their corresponding phenotypes. A new probability matrix Pm_*i*+1_ is then estimated from *G*sub_*i*_. The cycle of estimation, sampling, and selection is repeated until a stopping condition is met.

Based on elementary statistics, the probability of a genotype in *G*_*i*_, for some *i*, to be in the next generation (*i* ← *i* + 1) after sampling Pm_*i*_ is derivable from Pm_*i*_. If a prospective genotype of high quality (e.g., fitness value) has less probability of persisting to the next generation, it is less likely to be in the next generation. This EDA drawback was remedied in [43]. Another drawback of EDA is that after several generations, diversity of genotypes in *G*_*i*_, for large *i*, will be lost; a drawback is remedied in [44].

In Figure 3, there is consideration of the visually detected high (of blue hue) probability block of genes indexed 15 to 17 with task IDs 5, 15, and 17, respectively. During the sampling of the probability matrix depicted in the figure, task IDs that correspond to these high probability blocks are more likely to appear at gene indices of the offspring genotypes that correspond to the blocks, thereby preserving the blocks.

#### 2.5.1. EDA in Scheduling

In [9, 45], EDA was applied to solve an RCPS problem in a static environment with tasks on various execution modes. The authors utilized a probability matrix

(6) $$\begin{array}{c} \text{P}{\text{m}}_{i}=\left[\lambda_{j,\;k}\right], \end{array}$$

where *j* is the task index, *k* is the gene index of genotype used in EDA, and *i* is the EDA generation/cycle count resembling that in Algorithm 2. Before the start of the first generation (*i* = 0) in the applied EDA, all entries of Pm_0_ were set to 1/*N* where *N* is the number of tasks in the environment and is the genotype length. This implies that all of the tasks have equal probability of being placed into any gene location in any genotype formed during sampling of Pm_0_ at the first generation.

A copy *Pcp*_*i*_ of Pm_*i*_ was used to generate a genotype as follows: the *k*th column of *Pcp*_*i*_, that is, *Pcp*_*i*_(:, *k*) ≡ {*λ*_*j*,*k*_ | 1 ≤ *j* ≤ *N*} is sampled to obtain a task that can be assigned to the gene indexed *k* of the genotype. However, it could happen that not all tasks with nonzero corresponding probability in *Pcp*_*i*_(:, *k*) are eligible for the assignment due to the task precedence constraint in the RCPS problem. To remedy this, let *E*_*k*_ be a set of indices of eligible tasks with nonzero corresponding probability in *Pcp*_*i*_(:, *k*). Corresponding probabilities of these eligible tasks are provisionally revised to

(7) $$\begin{array}{c} {\hat{\lambda }}_{j,k}=\frac{\lambda_{j,k}}{\sum_{j\in E_{k}}\lambda_{j,k}}, \end{array}$$

not for updating entries of *Pcp*_*i*_, where *j* ∈ *E*_*k*_. The provisional probability matrix $\left\{{\hat{\lambda }}_{j,k}|j\in E_{k}\right\}$ is then sampled to obtain a task that will be assigned to gene indexed *k*. After this assignment, all entries of row *j* of *Pcp*_*i*_ are set to zero to avoid reassigning the obtained task thereafter. Further, each column of *Pcp*_*i*_ is renormalized. The steps described in this paragraph enable the assignment of a task to one gene only. They are repeated for each gene, from first (*k* = 1) gene to last (*k* = *N*), consecutively, thereby creating one genotype in the next generation genotype set *G*_*i*+1_ (step 2 of Algorithm 2).

To produce other genotypes in *G*_*i*+1_, the steps described in the last paragraph are repeated but starting with a fresh copy *Pcp*_*i*_ of *Pm*_*i*_. The remaining steps in Algorithm 2 are executed to complete one cycle.

#### 2.5.2. Other EDA Applications

EDA was applied in [46] to improve the performance of a technique, based on* reinforcement learning* (RL) [47], for solving a multiobjective problem. In this work, a probability matrix was revised every time the technique's RL system dynamically interacted with an environment. EDA was applied to classify tissues at molecular level in [48]. The selection of genotypes (an implementation of step 3 in Algorithm 2) in this work is based on the Pareto ranks and crowding distances [41] of their corresponding phenotypes.

EDA was applied in the optimization of a simple function with time-varying dimension in [24, 25]. In this application, the utilized probability matrix was a mixed Gaussian model whose number of clusters was determined through the Bayesian information condition. EDA was applied to other dynamic optimization problems with fixed dimensions [44, 49, 50]. In [49], some parameters of the current state of a dynamic environment were used to retrieve, from a memory, parameters of probability matrix utilized for solving a problem set in a previous state of the environment. These retrieved parameters were utilized to form the probability matrix of an EDA process that solved the problem set in the current state of the environment. Correction to population diversity loss in the EDA process was applied.

## 3. Materials and Methods

This section presents a test environment that sets some instances of the Φ^2^ problem solved by each technique in *T*. Further, it explores these techniques.  Section 3.1 provides information on the intuitive descriptions of the Φ^2^ problem. The formal definition of this problem is in Appendix A.  Section 3.2 describes the various instances of the Φ^2^ problem from which the dynamics of McBAR's performance for solving these instances are demonstrated.  Section 3.3 presents the technique referred to as* centroid-based adaptation with random immigrant* (CBAR) which is the precursor of McBAR.  Section 3.4 provides information on McBAR.  Section 3.5 elaborates our approach in applying EDA to the Φ^2^ problem. Further, it describes other techniques used to achieve goal 1 in the Introduction.

### 3.1. Intuitive Description of the Φ^2^ Problem

The selection of Φ^2^ as the test problem in this paper is based on the popularity of RCPS in the study of adaptation in dynamic environments [4]. The problem Φ^2^ is set in a military operation environment such that it is referred to as the military mission scheduling (MMS) problem. MMS is a process by which the goals of a commanding officer will reach fruition. For an extensive review on MMS, one may refer to the work of [6]. Intuitive descriptions of Φ^2^ are given in this section with examples. In this section, the military operation environment that sets problem Φ^2^ is simply referred to as the environment or the dynamic environment (to stress its variability).

#### 3.1.1. Notation

Consider the dynamic environment in which a snapshot is taken of its original state and at every moment it changes state. For example, a snapshot is taken of the environment that involves a valley occupied by rebels that pose a certain scheduling problem. Then, the next snapshot is taken when the environment changes to a state that, in addition to its last state, has a road-blocking landslide which hampers transport of logistics from a depot to the battlefield in the environment. This next snapshot poses a different scheduling problem. Note that a snapshot of the dynamic environment is a static environment.

Let *ϕ*_*i*_^2^ be a biobjective RCPS problem where schedule cost and duration are the conflicting objectives to minimize. Further, let this problem be set in a snapshot of a dynamic environment changing state for the *i*th time. Note that since a snapshot is a static environment, then *ϕ*_*i*_^2^ is a static problem. Let the integral-valued index *i* in *ϕ*_*i*_^2^ denote the* sequential order of state alteration* (SOSA) of the dynamic environment. For instance, third SOSA denotes the third moment the environment is changing state. Let zeroth SOSA correspond to the original state of the environment. Considering that the snapshot of the dynamic environment is taken immediately after this environment changes state, then the sequential order of taking the snapshot is similar to SOSA.

The dynamic RCPS problem Φ^2^ is viewed as a sequence of static problems *ϕ*_*i*_^2^; that is,

(8) $$\begin{array}{c} \Phi^{2}=\langle \phi_{0}^{2},\phi_{1}^{2},\ldots ,\phi_{i}^{2},\ldots ,\phi_{L}^{2}\rangle , \end{array}$$

where 〈 〉 denotes ordered set; subscripts *i* denote SOSA; and *L* denotes the number of moments at which the environment changes state. From here onwards, *ϕ*_*i*_^2^ will be referred to as subproblem of Φ^2^. In addition, the index *i* in *ϕ*_*i*_^2^ has the range 0 ≤ *i* ≤ *L*, assumed from here onwards. Intuitive descriptions of the *ϕ*_*i*_^2^ subproblem are given first before its formal definitions, found in Appendix A. These intuitive descriptions are as follows.

#### 3.1.2. Inputs

Tasks in the environment are characterized by the following.

1. task ID,
2. type of performed activity,
3. duration,
4. status,
5. number of items of a specified type of resource utilized,
6. precedence relationship to other tasks,
7. starting time.

The status of each of the tasks can either be ongoing, finished, or not yet executed. Canceled unexecuted tasks are not considered in this paper. The input information to the *ϕ*_*i*_^2^ subproblem is items 1 to 6 of each of the tasks in the environment. Some features of the tasks in the environment are found in Table 1. The first and second columns in this table are task IDs and durations, respectively.

The precedence relationships of tasks can be expressed as a network of nodes, which represent tasks, and arcs, which represent precedence relationships. An example network is illustrated in Figure 1. Now, suppose that, in Table 1, task 2 is to transport weapons and ammunition, task 16 is to interrogate prisoners of war (POWs), and task 17 is to clear a bombed area. Thus, the network in Figure 1 expresses the concept that the bombing of enemies must be done after the transportation of weapons and ammunitions and before clearing the bombed area and the interrogation of POWs.

Resources such as soldiers, fuel, and weapons are assets that can be utilized for a system to function. In RCPS, each type of resource has a limited number of items, a number that is input information to the *ϕ*_*i*_^2^ subproblem. The third to the last columns of in Table 1, respectively, are the resources *R*_1_ to *R*_4_ utilized by the tasks with IDs in the first columns. For instance, task 12 is to bomb enemies for 16 time units and to utilize at most four light mortar batteries (*R*_1_).

After one task is finished, resource items it had utilized are transferred to another task that will utilize all or some of the items or are returned to a depot called central base. Another input to the *ϕ*_*i*_^2^ subproblem is the cost of moving each resource item from one task location to the next. Task activity location is identified by the ID of the task. For instance, location 7 in a battlefield is where the task with ID 7, for example, to care for refugees by one infantry company, is executed. Central base has a location label of zero.

The transfer status of resource item can either be moved or unmoved. And, the availability-to-task status of resource item can either be available, broken/dead, or occupied (meaning utilized by a task). Additional inputs to the *ϕ*_*i*_^2^ subproblem are items' transfer and the availability status of resource items and the execution locations of tasks.

#### 3.1.3. Objective Functions

Minimization of schedule cost and duration are the two objectives in *ϕ*_*i*_^2^. They are mathematically defined in Appendix A. As pointed out in Introduction, these objectives are conflicting.

#### 3.1.4. Constraints

One constraint in the *ϕ*_*i*_^2^ subproblem is implied by the precedence network of tasks in the environment; namely, the starting time of any task in this network must be later than or equal to the longest end time of its predecessors. For example, based on Table 1, task 3 has duration of 16 time units. Based on Figure 1, if task 3 starts at five time units then, to satisfy the constraint, task 8 must start on or after 21 time units.

As mentioned above, *ϕ*_*i*_^2^ is an RCPS type of subproblem. Thus, in *ϕ*_*i*_^2^, the number of items of any type of resource utilized by all ongoing tasks in the environment is constrained. For example, there must be at most five C130 airplanes (*R*_3_ resource type) that can be used simultaneously at any instant during the military operation in the environment. This constraint is denoted by the column heading *R*_3_ ≤ 5 of Table 1.

#### 3.1.5. Output

**Figure 4:**
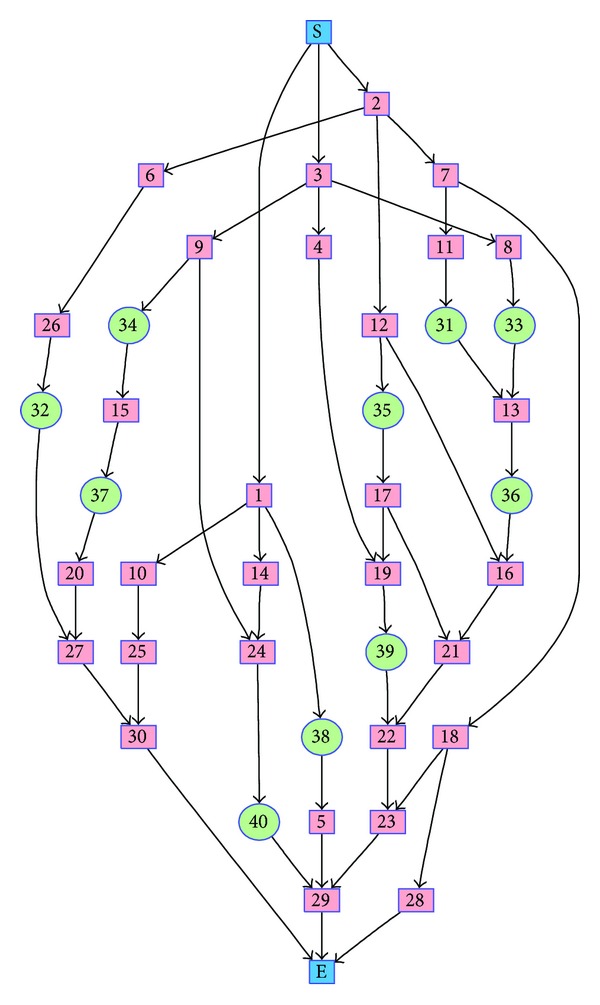
New task precedence labeled TNIS *T*_4_.

Some computational products of solving the *ϕ*_*i*_^2^ subproblem are genotypes, each expressed as an ordered set

(9) $$\begin{array}{c} I=\langle I_{1},I_{2},\ldots ,I_{k},\ldots ,I_{N}\rangle , \end{array}$$

where *I*_*i*_s are the ID of tasks in the environment, *k* is the gene index, and *N* is the genotype length. Note that any genotype mentioned from here onwards has a form similar to that in (9). The ordering of IDs in the genotype is based on a given task precedence network. The ordering rule is the following: any ID *I*_*k*_ in the genotype must correspond to a task whose successors do not have IDs *I*_*j*_ where *j* < *k*. For example, the section 〈1,14,2, 7,3, 9〉 of a genotype satisfies the ordering rule based on Figure 4. Any genotype which satisfies the ordering rule is characterized as task-precedence feasible. Genotypes are generated either through SSGS (explained in Section 2.1.2) or by some other means, such as described in Section 3.3.

Some more computational products of solving *ϕ*_*i*_^2^ are the starting times of tasks in the environment. These starting times are determined either through SSGS or by some other means, such as described in Section 3.3.4. Any solution to the *ϕ*_*i*_^2^ subproblem is an ordered set of the tasks with determined starting times and is referred to as a schedule,

(10) $$\begin{array}{c} S=\langle T_{1},T_{2},\ldots ,T_{k},\ldots ,T_{N}\rangle , \end{array}$$

where task *T*_*k*_ has ID *I*_*k*_ in (9). Based on the above-mentioned ordering rule of IDs in any genotype, every task *T*_*k*_ in (10) must not have successor *T*_*j*_ where *j* < *k*.

**Figure 5:**
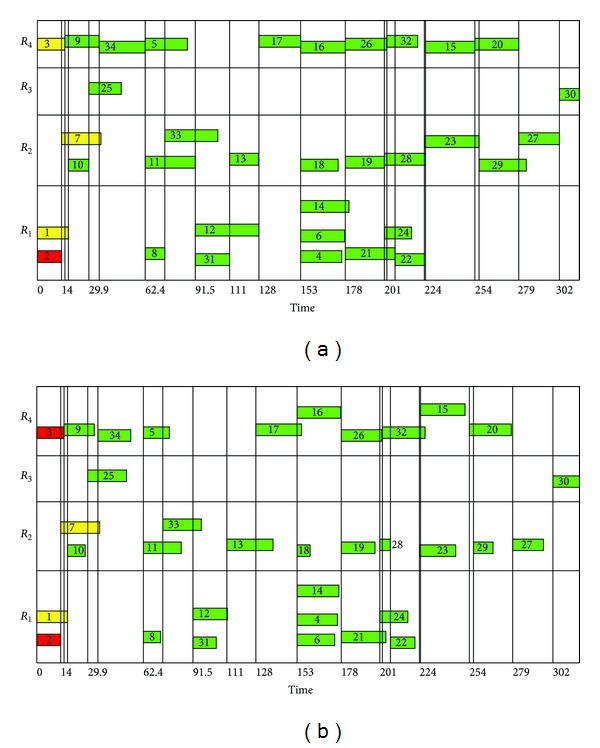
Solution to the subproblem set in the (a) fifth (b) sixth snapshot.

Sample schedules are depicted in Figures 5(a) and 5(b) where the horizontal axis is time; filled rectangles represent tasks whose task IDs are the labels of the rectangles; and the lengths of the rectangles denote the duration of the tasks that correspond to them. All rectangles inside the region labeled *R*_*i*_ correspond to tasks that utilize resources of similar label *R*_*i*_. All tasks in the schedules obey the task precedence relationship enshrined in Figure 4. For instance, in Figure 5(a), task 2 ends at 13 time units and task 7 starts at that similar time. Hence, task 7 succeeds task 2, thereby maintaining the task precedence relationship. More details of Figures 5(a) and 5(b) will be given in the next section.

#### 3.1.6. Dynamic Factors of Φ^2^

Sections 3.1.2 to 3.1.5 described the static subproblem *ϕ*_*i*_^2^ of Φ^2^. Let us now describe the features of the dynamic Φ^2^. In the above-mentioned military mission environment that sets Φ^2^, it could happen that the estimated completion duration of a military task will not be obeyed due to difficulties in terminating enemies. Thus, the task completion duration could vary from one snapshot of the environment to the next. The dynamic duration *d*_*j*_′ of any task with ID *j* in the environment is modeled as

(11) $$\begin{array}{c} d_{j}^{\prime }=Nm\left(d_{j},\delta \right)+\delta , \end{array}$$

where *Nm* is the normal distribution with the mean as the predefined task duration *d*_*j*_ (e.g., those listed in Table 1) and *δ* as the standard deviation.

It could also happen that the availability status of resources in the environment will change due to such factors as the fatigue of soldiers, the breakdown of equipment, and the delay in logistics. Further, it could also transpire that enemies retreat to recuperate and then attack at some later time. Consequently, new tasks, not accounted for in an original plan (a plan prior to any change in the environment), could be elicited. Thus, the total number of different tasks in the environment can change. The total number of tasks can only increase or be constant. Further, the number of finished tasks does not reduce the total number of tasks and there are no canceled unexecuted tasks.

To recapitulate, the availability status of resources used, duration, and total number of tasks are the dynamic factors in Φ^2^. To exemplify certain dynamics of Φ^2^, consider Figures 5(a) and 5(b) again. The schedule depicted in Figure 5(a) is one of the solutions to the *ϕ*_5_^2^ subproblem set in the snapshot taken at the fifth SOSA of the environment and at 13 time units. The schedule depicted in Figure 5(b) is one of the solutions to the *ϕ*_6_^2^ subproblem set in the snapshot taken at the sixth SOSA of the environment and at 16 time units. Each task in the schedule depicted in Figure 5(b) has a duration that is an alteration, following (11), of the duration of its pair task of similar IDs in the schedule depicted in Figure 5(a). The starting times of the pair of tasks are identical but their durations may not be so due to the dynamics of the environment. This is true for all pairs of tasks from the figures with similar IDs. The four tasks, with IDs 31 to 34, had already been added to the 30 tasks, with IDs 1 to 30, in the original plan.

In Figures 5(a) and 5(b), the darkest-background (red-background) rectangles correspond to finished task, for example, task 2; lightest-background (yellow) to ongoing tasks, for example, tasks 1 and 7; and the other colored rectangles to tasks yet to be executed. Based on Table 1 and on the task precedence in Figure 4, at 13 time units (which corresponds to Figure 5(a)), tasks 1, 3, and 7 are still ongoing, task 2 is finished, and the rest are yet to be executed. Further, at 16 time units (which corresponds to Figure 5(b)), tasks 1 and 7 are still ongoing, tasks 2 and 3 are finished, and the rest are yet to be executed.

### 3.2. Subproblem Instances *P*_*i*_^2^

Section 3.1 provides an intuitive description of the general features of the Φ^2^ problem, while Appendix A presents specific details of these features. This section provides information on the parametric values and categorical types of the features. Let problem Φ^2^ with concrete features be referred to as a problem instance. Different problem instances of Φ^2^ could be set in different environments, for example, scheduling problems for sea and land battles being expectedly different.

Section 3.2.1 provides information on problem instances of Φ^2^, information such as the types of resources utilized by the tasks and the number of new tasks at every SOSA of an environment.  Section 3.2.2 defines the functions used to obtain the concrete features of a problem instance given some parameters.  Section 3.2.3 gives details of the several computer simulations of the instances of Φ^2^.  Section 3.2.4 defines averages useful for describing the dynamic performances and for solving the problem instances, of techniques in *T* (defined in Section 3.5.2).

#### 3.2.1. Instances of Φ^2^ Problem

Problem instance *P* ^2^ of Φ^2^ is expressed as

(12) $$\begin{array}{c} P^{2}=\langle P_{0}^{2},P_{1}^{2},\ldots ,P_{i}^{2},\ldots ,P_{L}^{2}\rangle , \end{array}$$

where *P*_*i*_^2^ is an instance of *ϕ*_*i*_^2^ which, based on (8), is a subproblem of Φ^2^ set in the *i*th snapshot of an environment that has *L* number of moments of changes. Note that there could be several simultaneous changes in the environment at a single moment of changes. As a sample notation of subproblem instance, 12_7_^2^ denotes a subproblem of the instance, labeled 12, of problem Φ^2^ set in the seventh snapshot of the environment. Based on the ordered set 〈·〉 in (12), 12_7_^2^ also denotes the seventh subproblem of instance labeled 12 of problem Φ^2^. Any environment mentioned in this section is one that sets the *P* ^2^ problem instance and will be referred to as the environment.

Each task in the environment utilizes only one of the following four types of resources:

- light mortar batteries (*R*_1_) ≤16;
- infantry companies (*R*_2_) ≤17;
- C130s (*R*_3_) ≤5;
- Apache helicopters (*R*_4_) ≤16.

The constraint on the number of items of any listed resources that can be utilized by all ongoing tasks in the environment is indicated beside the resource label *R*_*i*_. Note that the availability status (defined in Section 3.1.2) of any of the resources can change from available to occupied (e.g., mortars firing) or vice versa (e.g., mortars returned from combat to depot) in the course of executing a schedule, that is, without the effects of the environmental dynamics. The only effect of the environment on the availability status, being proposed in this paper, is to change this status to broken (e.g., killed infantry soldier) from being available or occupied.

As mentioned in Section 3.1.2, properties of the tasks in the environment are found in Table 1. The environment has 30 original tasks (existing in the environment before it is changed) that have precedence relationship as depicted in Figure 1. A total of ten new tasks are added to the original tasks during the entire dynamics of the environment. New *dN* tasks, that occur in a particular SOSA of the environment, have IDs *I*, *l* < *I* ≤ *l* + *dN*, where *l* is the maximum task ID prior to this SOSA. For example, if, at the last SOSA, the maximum task ID is 30 and there are four new tasks added at the current SOSA, then these new added tasks will have IDs 31 to 34.

The considered types of changes in the environment are listed in Table 2. The first column contains labels of types of changes, and the second contains the parameters that change simultaneously. For example, type 6 of the changes is a type of change involving changes in the environment where task duration, total number of tasks, and resource availability change simultaneously.

The types of changes are chained to form a sequence of changes. Each* type of sequence of changes* (TSC) is presented as a column, labeled *S*_*i*_, in Table 3. The first and last column of this table contain, respectively, the SOSAs and times at which specific type of changes occur. For example, sequence *S*_3_ begins and followed by changes in task duration only (type 0 in Table 2). The next type of change in this sequence is simultaneous changes in task duration, total number of tasks, and resource availability (type 6 in Table 2) that occur at the third SOSA of the environment. The number of moments at which the environment changes state is *L* = 12.

Some components of each TSC are types of changes that involve an increase in the total number of tasks. For example, *S*_3_ has types of changes 6, 5, 2, and 3 that occur, respectively, at third, eighth, tenth, and 12th SOSAs of the environment and all involve an increase in the total number of tasks, based on Table 2.* Task number increase sequence* (TNIS), labeled as *T*_*k*_ in Table 4, is a sequence of the numbers of new tasks that appear at some SOSAs of the environment. The sequence order of these numbers is the order of appearance of the batches of new tasks. Using the example above, if *S*_3_ has TNIS of *T*_5_, there will be five, three, one, and one new tasks that appear at the third, eighth, tenth, and 12th SOSAs of the environment, respectively, based on Table 4. The first column of Table 4 is the order at which the batch of new tasks appear. Note that even with similar TSC but with different TNIS the number of new tasks, in a given SOSA of the environment, could be different.

**Figure 6:**
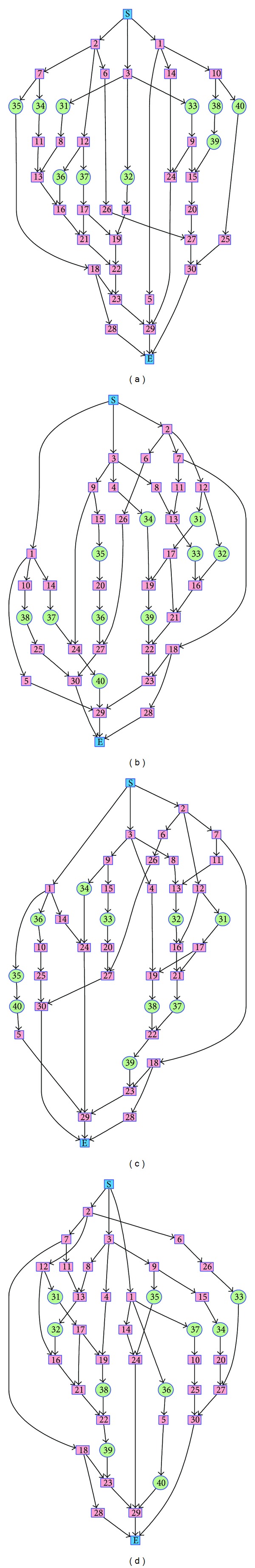
TNIS for (a) *T*_3_ (b) *T*_5_ (c) *T*_6_ (d) *T*_7_.

Task precedence networks that correspond to TNIS of *T*_3_, *T*_5_, *T*_6_, and *T*_7_ are illustrated in Figures 6(a) to 6(d), respectively, while that of *T*_4_ is in Figure 4. They are formed by placing the new tasks that appear in the environment to the original task precedence network, illustrated in Figure 1, and differing in their forms by the locations in which the new tasks are placed.

Some components of the sequences of changes in Table 3 involve change in task duration. The amount of change in task duration is modeled by (11) whose *δ* has to be specified. To recapitulate, a *ϕ* ^2^ instance is defined by a particular sequence (e.g., *S*_3_) of changes in the environment, subsequence (e.g., *T*_5_) of increases in the total number of tasks in the sequence, and the value of *δ* in (11).

Table 5 lists instances of Φ^2^ labeled from 1 to 30. With the column of types of TNIS as the reference column, the instances with labels at the left and right correspond to *δ* = 3.0 and *δ* = 6.0, respectively; instances with labels under *S*_*i*_ have TSC of *S*_*i*_; and instances with labels at the same row as *T*_*k*_ have TNIS of *T*_*k*_. For example, instance 25 has TSC of *S*_1_, TNIS of *T*_7_, and task duration changes modeled by (11) with *δ* of 3.0.

#### 3.2.2. Tables as Functions

Let us define some functions that yield values in Tables 2 and 4 and which are useful in the succeeding sections. Given a problem instance labeled *Ns*, a TSC *S*_*i*_ can be obtained using Table 5. Given an SOSA *S* of an environment that sets *Ns*, a change type label *C*type can be determined using *S*_*i*_ in Table 3. Thus, Tables 5 and 3 serve as a function *Ct*,

(13) $$\begin{array}{c} C\text{type}=Ct\left(Ns,S\right). \end{array}$$

Using *C*type in Table 2, the environmental attributes that change simultaneously can be determined. For example, instance 3 has TSC of *S*_3_, based on Table 5. Based on the *S*_3_ column of Table 3, the change type at the eighth SOSA of the environment is 5; that is, *Ct*(3,8) = 5. Based on Table 2, type 5 of changes signifies simultaneous changes in resource availability and the total number of tasks in the environment.

Table 5 serves as a function *N*type to map an instance label to type of task-precedence network,

(14) $$\begin{array}{c} N\text{type}=Pt\left(Ns\right). \end{array}$$

Note that, based on the *S*_3_ column of Table 3, the total number of tasks increases for the 3rd occasion at the eighth SOSA of the environment. Further, *T*_3_ = *Pt*(3); that is, instance 3 has TNIS of *T*_3_. The intersection of the third row, which is the third occasion order, and *T*_3_ column in Table 4 is two, the number of new tasks appearing at the eighth SOSA of the environment. Thus, the set of Tables 2, 4, and 5 serve as a function *Nt* to determine the number of new tasks,

(15) $$\begin{array}{c} N\text{tasks}=Nt\left(Ns,S\right). \end{array}$$

This function is applicable only when *Ct*(*Ns*, *S*) yields a type of changes that is 2, 3, 5, or 6 which, based on Table 2, all involve an increase in the total number of tasks. Otherwise, there is no new task at a given *S*th SOSA of the environment.

For convenience in the succeeding discussions, let the functions *Nt*, *Pt*, and *Ct* be distributive to vector elements; that is, if *Ns* = 〈*x*_1_, *x*_2_,…, *x*_*n*_〉,

(16) Ctype=Ct(〈x1,x2,…,xn〉,S)=〈Ct(x1,S),Ct(x2,S),…,Ct(xn,S)〉,

(17) Ntasks=Nt(〈x1,x2,…,xn〉,S)=〈Nt(x1,S),Nt(x2,S),…,Nt(xn,S)〉,

(18) Ntype=Pt(〈x1,x2,…,xn〉)=〈Pt(x1),Pt(x2),…,Pt(xn)〉.

For example, for an ordered set of instance labels 〈1,7, 13,19,25〉, *Ct*(〈1,7, 13,19,25〉, 4) = 〈2,2, 2,2, 2〉.

#### 3.2.3. Simulations

The environment is computer simulated whereby, to reflect a real-life scenario, its resources may be moved from one location of its task to another (e.g., tanks are moved from base camp to a valley); the status of its tasks and resources (enumerated in Section 3.1.2) may be altered (e.g., soldiers killed); and its type of dynamics (e.g., *S*_2_) is implemented. The environment is simulated for several times where, in each simulation,

1. the original (before any change in the environment) durations of unfinished tasks in the environment are added with random samples of the model in (11). Consequently, an unfinished task in the snapshot taken at the *i*th SOSA of one environment simulation could differ from the duration of the task with same ID in the snapshot taken at the same SOSA of other environment simulations. For example, if task 43, in the fifth snapshot of the second environment simulation, has a duration of 16 time units, its duration can be 25 time units in the fifth snapshot of the ninth environment simulation. The difference could be true for all unfinished tasks in all snapshots taken from the first to the *L*th SOSA of all environment simulations. All other features (e.g., number of in-use resources) of the environment are identical across simulations;
2. subproblem instances are sequentially solved (e.g., from *P*_0_^2^ to *P*_*L*_^2^) independently by every technique in *T*. Further, the environment is simulated, at its *i*th SOSA, before solving the subproblem instance *P*_*i*_^2^ (*P* could be any given instance label) set in this environment. Note that despite differences between elements of a set of the subproblem instance simulations (due to the addition of different random values to duration of tasks in different simulations), each technique in *T* solves this same set. This approach will reduce the number of variables to consider in comparing the techniques;
3. The random seed used, by the evolutionary processes of techniques in *T*, to solve *P*_*i*_^2^ subproblem, 0 ≤ *i* ≤ *L*, set in a simulation of the environment, could differ from that of the other simulations of the environment.

#### 3.2.4. Averages

Let us discuss the types of averages utilized to analyze the performances of techniques in *T*. The average **E**_*d*_^*δ*^[dSC(*A*, *B*)] differential set coverage of technique *A* over technique *B* will be used to compare the performances of these techniques in solving subproblem instances of Φ^2^, at different values *d* of *δ*. The parameter *δ* is found in (11) which models the change in duration of tasks in environments that set the instances. The average **E**_*d*_^*δ*^[dSC(*A*, *B*)] is defined as

(19) Edδ[dSC(A,B)]=∑i=1Ns1Ns∑j=1Nc1Nc∑k∈In⁡(δ)1|In⁡(δ)|dSC(A,B,i,j,k),

where dSC(*A*, *B*, *i*, *j*, *k*) is the differential set coverage dSC(*A*, *B*) (defined in (4)) of technique *A* over technique *B* determined for subproblem instance *k*_*j*_^2^ (in instance with label *k* in Table 5) and at the *i*th simulation of this instance. Further, *Ns* is the number of simulations and *Nc* is the number of subproblems in Φ^2^, including *ϕ*_0_^2^. Furthermore, In⁡(*δ*) is a set of labels of instances in Table 5 that utilize a given value of *δ*; for example, based on Table 5, In⁡(3.0) = {1,2, 3,7, 8,9, 13,14,15,19,20,21,25,26,27}.

The average **E**_*t*_^*τ*^[dSC(*A*, *B*)] differential set coverage of technique *A* over technique *B* will be used to compare the performances of these techniques at different types *t* of changes listed in Table 2. It is defined as

(20) Etτ[dSC(A,B)]  =∑i=1Ns1Ns∑k=1Nn1Nn∑j∈Soc(k,τ)dSC(A,B,i,j,k)|Soc(j,τ)|,

where *Nn* = 30 is the number of all instances in Table 5; Soc(*k*, *τ*) is the set of SOSAs of the environment that sets instance labeled *k* at which type *τ* of changes occurs. To exemplify, consider instance labeled 1 which, based on Table 5, has TSC *S*_1_. In this TSC, SOSAs with type *τ* = 0 of changes (task duration change only, based on Table 2) are in the set Soc(1,0) = {1,2, 3,5, 9,12}, based on Table 3. Note that zeroth SOSA is excluded in the definition of the averages.

The average **E**_*j*,*k*_^*σ*^[dSC(*A*, *B*)] differential set coverage of technique *A* over technique *B* will be used to compare the performances of these techniques at different simulations of subproblem instances of Φ^2^. It is defined as

(21) $$\begin{array}{c} E_{j,k}^{\sigma }\left[\text{dSC}\left(A,B\right)\right]=\frac{\sum_{i=1}^{Ns}\text{dSC}\left(A,B,i,j,k\right)}{Ns}, \end{array}$$

where all indices are already defined in this subsection.

Note that all of the averages are derived from differential set coverage which, as mentioned in Section 2.3, is a measure of the performance of one technique over another. We then take the following: if any of the averages is greater than zero, then technique *A* performs better than technique *B*, in determining solutions to instances of Φ^2^ listed in Table 5, over the domain at which this average is taken.

### 3.3. Centroid-Based Adaptation with Random Immigrants (CBAR)

McBAR is an extension of the memory-based EA technique referred to as* centroid-based adaptation with random immigrant* (CBAR). CBAR was applied in [6] to solve a class Γ^2^ of biobjective dynamic RCPS problems with a fixed total number of tasks. Γ^2^ is viewed as a sequence of the static RCPS subproblems *γ*_*i*_^2^ and each has schedule cost and duration as objectives to minimize

(22) $$\begin{array}{c} \Gamma^{2}=\langle \gamma_{0}^{2},\gamma_{1}^{2},\ldots ,\gamma_{i}^{2},\ldots ,\gamma_{L}^{2}\rangle , \end{array}$$

where *i*, as in Section 3.1.1, is the index of a snapshot taken at the *i*th SOSA of an environment that sets Γ^2^. From this point to Section 3.3.6, the environment that sets Γ^2^ is simply referred to as the environment or the dynamic environment.

Being an implicit memory-based technique (explained in Section 2.2), CBAR utilizes representatives of sets of solutions to past subproblems *γ*_*n*_^2^ to compute the solutions to the current subproblem *γ*_*c*_^2^, where *n* < *c*. Each representative is the centroid of genotypes that correspond to nondominated solutions to the *γ*_*n*_^2^ problem. A centroid *C*(*t*) is a genotype whose *k*th gene is [6]

(23) $$\begin{array}{c} c_{k}\left(t\right)=\left\lfloor \frac{1}{N_{d}}\underset{x^{j}(t)\in \text{Gnds}(t)}{\sum }x_{k}^{j}\left(t\right)\right\rfloor , \end{array}$$

where *k* = 1,…, *N*_*t*_; *N*_*t*_ is the total number of tasks in the snapshot taken at the *t*th SOSA of the environment; *x* ^*j*^(*t*) is a genotype that corresponds to a schedule as a solution to the *γ*_*t*_^2^ problem; *x*_*k*_^*j*^(*t*) is the *k*th gene/ID in the *x* ^*j*^(*t*) genotype (defined in (9)); Gnds(*t*) is a population of genotypes that have one-to-one correspondence to all solutions in the nondominated set Pnds(*t*) of solutions to the *γ*_*t*_^2^ subproblem; and ⌊·⌋ is the operator to round its real-value argument to an integer. Note in (23) that the equally-weighted average (mean) of IDs *x*_*k*_^*j*^(*t*) forms centroid genes.

#### 3.3.1. Centroid Repair

Centroid *C* formed through (23) may not necessarily be task-precedence-feasible, as defined in Section 3.1.5. If this is so, it will be repaired. Before explaining the repair process, a definition will be presented. Given a genotype *R*, its complementary genotype *R* ^*c*^ is *C* less the elements of *R*. For example, let the genotype *R* = 〈2,1〉 and the centroid

(24) $$\begin{array}{c} C=\langle 14,1,2,7,9,3,\ldots \rangle , \end{array}$$

where numbers to the right of 3 are fixed but not shown for brevity. Thus, *R* ^*c*^ = 〈14,7, 9,3,…〉. The repair of centroid *C* is undertaken by successively appending (to be described below) IDs to a genotype *R* which starts from empty. The ID *ρ* from *C* will be appended to *R* if it satisfies the following appending rule: the appending of ID *ρ* should result in a new *R* whose corresponding complementary genotype *R* ^*c*^ has IDs of tasks that are not predecessors of the task with ID *ρ* and *ρ* should not be found in the former *R*. However, if *ρ* does not satisfy the appending rule, a different ID *ρ*′ is randomly picked, which satisfies the appending rule from *R* ^*c*^ and then appended to the former *R*. Note that the complementary genotype used in checking the satisfiability of *ρ*′ to the appending rule is different from *R* ^*c*^ where *ρ*′ is picked.

At the start of the repair of centroid *C*, the first element of *C* is attempted to be appended to an empty genotype *R* following the process of appending described previously. Note that based on the explanation above, the element or a different element may be appended to *R*. Next, the second element of *C* is attempted to be appended to *R* (which has one element at this stage). This repair process is continued until the last element of *C* is attempted to be appended to *R*. After this stage, *R* is the repaired version of *C*. The following three paragraphs will provide examples of this repair process.

Consider the centroid *C* in (24) whose repair will be based on the task-precedence network in Figure 4. The above-mentioned repair process starts by attempting to append the first gene/ID 14 of *C* to an empty genotype *R*. So let the new *R* = 〈14〉 whose complementary genotype *R* ^*c*^ = 〈1,2, 7,9, 3,…〉. However, ID 1 in *R* ^*c*^ is the ID of the task that is the predecessor (based in Figure 4) of task with ID 14. Thus, the above-mentioned appending rule is violated. A random pick of ID, say 1, from *R* ^*c*^ is then undertaken. If 1 is appended to the former *R* (which is empty), the new *R* becomes 〈1〉 whose complementary genotype *R* ^*c*^ = 〈14,2, 7,9, 3,…〉. Now, no task with ID in the new *R* ^*c*^ is a predecessor (based in Figure 4) to the task with ID 1. Thus, the appending of 1 to *R* is allowed, thereby obtaining *R* = 〈1〉. Notice that, based on Section 3.1.5, the obtained *R* is task-precedence feasible.

The next step to the repair process is to attempt to append the second gene/ID 1 of the centroid *C* to *R*. Note that 1 is already present in *R* = 〈1〉 whose complementary genotype *R* ^*c*^ = 〈14,2, 7,9, 3,…〉. Thus, based on the above appending rule, a randomly chosen ID, say 14, is randomly picked from *R* ^*c*^. It is then appended to *R* to obtain *R* = 〈1,14〉 whose complementary genotype *R* ^*c*^ = 〈2,7, 9,3,…〉. Now, no task with ID in the last *R* ^*c*^ is a predecessor to the task with ID 14. Thus, the appending of ID 14 is allowed, thereby obtaining *R* = 〈1,14〉. Again, notice that this obtained *R* is task-precedence feasible.

Consider now the third gene/ID 2 of *C*. When it is appended to *R* = 〈1,14〉 yields *R* = 〈1,14,2〉 whose complementary genotype is *R* ^*c*^ = 〈7,9, 3,…〉. Now, no task with ID in *R* ^*c*^ is a predecessor to the task with ID 2. Thus, the appending of 2 is permitted. Again, notice that the obtained *R* = 〈1,14,2〉 is task-precedence feasible. The repair process is continued until the last gene of *C* is attempted to be appended to *R*. Based on this sample repair process, the resulting *R* at each appending cycle is task-precedence feasible. It is straightforward to prove that after *R* is completely appended, it is task-precedence feasible.

Consider the expressions of the completely appended *R*(*t*) = 〈*r*_1,*t*_, *r*_2,*t*_,…, *r*_*j*,*t*_,…, *r*_*N*_*t*_,*t*_〉 and the centroid *C*(*t*) = 〈*c*_1,*t*_, *c*_2,*t*_,…, *c*_*j*,*t*_,…, *c*_*N*_*t*_,*t*_〉, where *N*_*t*_ is defined in connection with (23) and *t* is a SOSA of the environment. The element *r*_*j*,*t*_ of *R*(*t*) could be viewed as the result of mapping the element *c*_*j*,*t*_ of *C*(*t*). Let this mapping be denoted by *R* and be referred to as* random repairer*. Formally stating the repair,

(25) $$\begin{array}{c} r_{j}\left(t\right)=\{\begin{array}{cc} c_{j}\left(t\right) & c_{j}\left(t\right)\;\text{satisfies}\;\text{append}\;\text{rule}\\ R\left(c_{j}\left(t\right)\right) & \text{otherwise}, \end{array} \end{array}$$

where *c*_*i*_(*t*) is defined in (23).

#### 3.3.2. Initial Population

One subalgorithm of CBAR is the creation of an initial population which CBAR evolves to obtain solutions to the *γ*_*t*_^2^, *t* ≥ 0, subproblem (defined in (22)) set in the *t*th snapshot of the environment. For *t* = 0, this initial population is a set of *N* SSGS-generated genotypes. For *t* > 0, it is expressed as

(26) $$\begin{array}{c} G_{0}\left(t\right)\equiv C\left(t\right)\cup \text{Rnd}\left(t\right)\cup \text{Ichs}\left(t-1\right), \end{array}$$

where

(1) *C*(*t*) is a set of centroids,
  (27) $$\begin{array}{c} C\left(t\right)=\overset{t-1}{\underset{k=t_{o}\left(t\right)}{⋃}}R\left(k\right); \end{array}$$
(2) *R*(*k*) is the repaired centroid of the population Gnds(*k*) of genotypes that corresponded to all the nondominated solutions to the *γ*_*k*_^2^ subproblem;
(3) The starting index of population *R*(*k*) is
  (28) $$\begin{array}{c} t_{o}\left(t\right)=\max\left\{t-N_{c},0\right\}; \end{array}$$
(4) *N*_*c*_ is a given maximum number of centroids in any initial population;
(5) Rnd(*t*) is a set of SSGS-generated genotypes, |Rnd(*t*)| = *N* − |*C*(*t*)| − 1;
(6) *N* is a fixed size of the initial population;
(7) Ichs(*t* − 1) is the chosen genotype in Gnds(*t* − 1).

Note that the definition of *t*_*o*_(*t*) in (28) restricts the maximum number of centroids in *C*(*t*) to *N*_*c*_. Recall from Section 2.1.2 that SSGS randomly selects IDs of eligible tasks, in a given RCPS environment, to form genotypes. Hence, these genotypes have a stochastic facet. Thus, the SSGS-generated set Rnd(*t*) in (26) constitutes the random/diversifying component of the initial population *G*_0_(*t*).

#### 3.3.3. Genetic Operators

Being an EA-based technique, CBAR has an evolutionary process. The crossover and mutation operators in this process are designed to suit our system. The designed crossover operator has the following algorithm. Consider two parent genotypes *G*_1_ and *G*_2_ for crossover from whom two offspring genotypes *O*_1_ and *O*_2_ are generated. Each of the parent genotypes is broken into three parts at two randomly selected crossing points labeled *L* and *R*. The crossing point *L* is between gene locations *l* and *l* + 1 and the crossing point *R* is between gene locations *r* and *r* + 1, where 1 < *l* < *r* < *N* and *N* is the length of the parent and offspring genotypes. These crossing points are similar for both parents. First, offspring *O*_1_ inherits the first part of parent *G*_2_. Second, it inherits the genes of parent *G*_1_ and consecutively places these genes at its gene locations *l* + 1 to *r*. Suppose that the *k*th gene location of *O*_1_ is to be filled with a gene, where *l* < *k* ≤ *r*. The genes of parent *G*_1_, located from one to *r*, are consecutively searched for a gene different to all genes in *O*_1_ located before location *k*. Once found, the search is stopped and the different gene is placed at location *k* of *O*_1_. Third, the second step is repeated with genes of *O*_1_, located from *r* + 1 to *N*, inheriting from *G*_2_ where the search process is applied to all genes of parent *G*_2_. The three parts of *O*_2_ are inherited by consecutively using parents *G*_1_, *G*_2_, and *G*_1_ in the first to the third steps, respectively. The presented inheriting process has similarities to the PMX crossover [51]. The mutation of a genotype swaps two of its consecutive genes, at a randomly selected gene location with predefined probability, provided that the resulting genotype is task-precedence feasible, as defined in Section 3.1.5.

#### 3.3.4. Schedule Formation

Let us discuss a method, referred to as* Schedule Formation*, for determining a schedule from a genotype. Given the genotype *G* = 〈*g*_1_, *g*_2_,…, *g*_*k*_,…, *g*_*N*_〉 of length *N* as described in Section 3.1.5, its corresponding schedule is formed through the following scheme. Each gene/ID in this genotype is used consecutively, from first to last, to determine the starting time of the task with this ID. At the stage of using the gene/ID *g*_*k*_, the starting time *t* of task with this ID is set to the earliest time later than or equal to

(29) $$\begin{array}{c} t=\max\left\{0,\max\left\{\text{s}{\text{t}}_{i}+d_{i}\;|\;i\in \text{Pred}\left(j\right)\right\}\right\}, \end{array}$$

where all terms in this equation are defined in Section 2.1.2, except *t*. After the consecutive usage of genes, the starting times of all tasks with IDs in the genotype are all determined. Based on the definition of schedule in Section 3.1.5, the schedule that corresponds to the genotype is obtained. The cost to implement this schedule is determined through ((A.8)) and its makespan is the end time of its last task to finish.

#### 3.3.5. The Algorithm of CBAR

Static subproblems in the dynamic problem Γ^2^ (expressed in (22)) are solved by CBAR sequentially, from *γ*_0_^2^ to *γ*_*L*_^2^, where *L* is the number of SOSAs of the environment. To determine solutions to the subproblem *γ*_0_^2^ (set in the original state of the environment), CBAR simply executes SSGS (explained in Section 2.1.2) to generate an initial population *G*_0_(0) of genotypes. CBAR then evolves *G*_0_(0) to obtain the population *G*(0) of evolved genotypes. Then by using the genotypes in *G*(0) for the Schedule Formation method described in Section 3.3.4, the population *P*(0) of baseline schedules is then determined. These schedules are solutions to the *γ*_0_^2^ subproblem.

The evolutionary process of CBAR is performed through NSGA-II. In each cycle of this process, NSGA-II creates an offspring genotype population from a parent genotype population. We let NSGA-II use the revised genetic operators described in Section 3.3.3 to create the offspring. The offspring genotypes are feed to the Schedule Formation method to obtain the cost and duration of the schedules that correspond to these genotypes. The Pareto rank and crowding distance for each of these schedules are determined based on its cost and duration [41]. Following the explanation in Section 2.4, NSGA-II uses the Pareto ranks and crowding distances of the schedules in its selection process to obtain its next generation genotype and schedule populations.

To determine solutions to the subproblem *γ*_*t*_^2^, *t* > 0, CBAR starts by determining the centroid *C*(*t* − 1) of genotypes that correspond to the nondominated solutions to the subproblem *γ*_*t*−1_^2^ (set in the last snapshot before the *t*th snapshot of the environment) through (23) then repairs this centroid using *R* (explained in Section 3.3.1), followed by forming an initial population *G*_0_(*t*) through (26), and then evolves this initial population using NSGA-II as described above. The schedules that correspond to the evolved genotypes are the solutions to the subproblem *γ*_*t*_^2^ and form a population *P*(*t*).

From here onwards, CBAR is designated as the one which generates the initial population *G*_0_(0) and the SSGS-generated genotype population Rnd(*t* − 1) in (26), at any *t* > 0, by having the SSGS algorithm as the generator of these genotypes and as the one which evolves the initial populations described in this section by having NSGA-II as its evolutionary engine.

#### 3.3.6. Chosen Schedule

After CBAR has computed the population *P*(*t*) of solutions to the *γ*_*t*_^2^ subproblem, a schedule *C* is chosen from *P*(*t*). This chosen schedule is utilized in the simulation of the environment. When, in this simulation, the environment changes state for the (*t* + 1)th SOSA, CBAR evolves an initial population *G*_0_(*t* + 1) to obtain the solutions to the *γ*_*t*+1_^2^ subproblem set in this state of the environment. The initial population contains, based on (26), the chosen genotype Ichs that corresponds to the chosen schedule *C*.

Consider the tasks, of a schedule that corresponds to a genotype in *G*_0_(*t* + 1) different to Ichs, whose IDs are similar to either on-going or finished tasks of the chosen schedule (corresponds to Ichs) at the instant of the (*t* + 1)th SOSA of the environment. The starting time of each of these tasks is set equal to its counterpart (task of same ID) in the chosen schedule. This process is performed on all schedules that correspond to genotypes in *G*_0_(*t* + 1) different to Ichs and also on offspring genotypes in every cycle of the evolutionary process of NSGA-II. At the end of the evolutionary process of NSGA-II, the evolved schedules have copies of the ongoing or finished tasks of the chosen schedule. Note that these evolved schedules, as solutions to the *γ*_*t*+1_^2^ subproblem (set in the current state of the environment), are revisions of the chosen schedule Ichs which is one of the solutions to the *γ*_*t*_^2^ subproblem (set in the last state of the environment). Thus, the preservation (copying) of the ongoing and finished tasks on the revised schedules demonstrates CBAR to abide by the schedule revision rule described in Section 2.1.

#### 3.3.7. Inapplicability of CBAR

Let us now discuss how CBAR becomes inapplicable for solving the dynamic problem Φ^2^ which involves change in total number of tasks. Suppose that a static subproblem *ϕ*_0_^2^ in Φ^2^ is set in an environment snapshot that has *N*_*t*_ total number of tasks. For this subproblem, a sample task precedence network is depicted in Figure 4 where original tasks (the only tasks found in the original state of the environment) and additional tasks are represented by numbered rectangles and circles, respectively.

Suppose that the total number of tasks is increased for the first time to *N*_*τ*_ = *N*_*t*_ + *dN* at the *τ*th SOSA of an environment that sets the *ϕ*_0_^2^ subproblem; *τ* > 0. When *dN* or more tasks are finished at the *τ*th SOSA of the environment, IDs of new *dN* tasks can be placed instead of the IDs of *dN* finished tasks in each genotype in the initial population *G*_0_(*τ*) used to solve the *ϕ*_*τ*_^2^ subproblem through CBAR. Thereby, the need to change genotype length may be avoided. However, in the case where there is no task finished at the *τ*th SOSA of the environment, the genotype must have a sufficient number of genes to accommodate IDs of *N*_*t*_ original and *dN* new tasks. Thus, this condition requires the genotype to be implemented with *N*_*τ*_ number of genes. Therefore, the nondominated set of solutions (schedules) to the *ϕ*_*τ*_^2^ subproblem must correspond to genotypes—forming the set *G*(*τ*)—each of length *N*_*τ*_.

Before continuing our discussion, let us take the notation,

(30) $$\begin{array}{c} G\left[t\right]=\overset{t-1}{\underset{k=t_{o}(t)}{⋃}}G\left(k\right), \end{array}$$

to denote the combined genotype populations that correspond to the nondominated sets of solutions to static problems *ϕ*_*t*_*o*__^2^ to *ϕ*_*t*−1_^2^, where *t*_*o*_(*t*) is defined in (28). Based on the definition of *t*_*o*_(*t*) and on (30), the number of genotype populations combined to form *G*[*t*] is limited at most to *N*_*c*_, the maximum number of centroids used in (28).

Suppose that there is no finished task prior to the *τ*th SOSA of an environment that sets the dynamic Φ^2^ problem, where 0 < *τ* < *N*_*c*_. Thus, each genotype in the combined populations *G*[*τ*] has *N*_*t*_ number of genes. Based on (26), repaired centroids and chosen genotype in the initial population *G*_0_(*τ*) are derived from *G*[*τ*]. Thus, they each have a length of *N*_*t*_. Considering that CBAR does not increase the length of each of the genotypes as it evolves them, then it cannot evolve *G*_0_(*τ*) (whose genotypes have the length *N*_*t*_) to produce *G*(*τ*) whose genotypes each must have the length *N*_*τ*_ > *N*_*t*_.

### 3.4. Mapping of Task ID for Centroid-Based Adaptation with Random Immigrants (McBAR)

**Figure 7:**
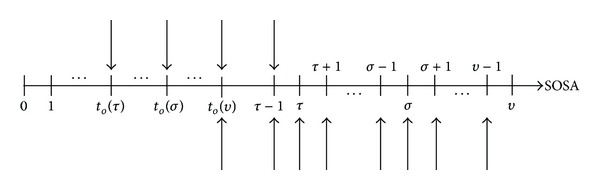
The SOSA of the environment at which genes are inserted.

Let us now consider how CBAR is revised to overcome its unsuitability to solve the dynamic problem Φ^2^ (defined in (8)). Let any environment mentioned in this section be the one that sets the Φ^2^ problem and be referred to as the environment or the dynamic environment. Now, suppose that new tasks (not found in the original state of the environment) appear for the first time at the *τ*th SOSA of the environment. Following [23], the partial remedy for the unsuitability is to insert new genes, that correspond to the new tasks, to each genotype in the population *G*(*k*) which correspond to the nondominated set *P*(*k*) of solutions to the static subproblem *ϕ*_*k*_^2^ where *t*_*o*_(*τ*) ≤ *k* < *τ* with *t*_*o*_(*τ*) as defined in (28). The insertions of the new genes to genotypes in populations *G*(*t*_*o*_(*τ*)) to *G*(*τ* − 1) are depicted in Figure 7 as downward-pointing arrows. In this figure, the horizontal line is the SOSA of the environment. Further, the *k*th SOSA at which arrow point corresponds to subproblem *ϕ*_*k*_^2^ whose nondominated solutions have corresponding genotypes that are being inserted with the new genes. Based on the range of *k*, subproblems encountered in the gene-insertion process are *ϕ*_*t*_*o*_(*τ*)_^2^ to *ϕ*_*τ*−1_^2^. These subproblems are set in the snapshots of the environment taken, respectively, from *t*_*o*_(*τ*) until before the first increase in the total number of tasks.

After the insertion of new genes into each genotype of *G*(*k*), *t*_*o*_(*τ*) ≤ *k* < *τ*, the initial population in (26) is formed and then evolved by CBAR. The evolved genotypes are inputted to the Schedule Formation scheme in Section 3.3.4 to obtain the solutions to subproblem *ϕ*_*τ*_^2^. The technique referred to as* gene-inserting CBAR* (GIBAR) is CBAR that includes executing the gene-insertion process every time new tasks appear in the environment. This nomenclature will be useful in Section 3.5.2.

Now, our previous work [14] showed the performance (solution-searching ability) of CBAR to be degraded by the gene insertion. The adopted resolution of this side effect is to map task IDs in each of the gene-inserted genotypes in *A*(*k*) to reduce the discontinuity of IDs along each genotype, where *A*(*k*) is the population of all gene-inserted genotypes of *G*(*k*). The mapping process is applied for each *A*(*k*), *t*_*o*_(*τ*) ≤ *k* < *τ*. To facilitate ease in succeeding discussions, let *B*(*k*) be the set of all ID-mapped gene-inserted genotypes of *A*(*k*). Note that *B*(*k*) is derived from *G*(*k*) which is a set of genotypes that correspond to the nondominated solutions of the *ϕ*_*k*_^2^ subproblem. Further, let the combined population be

(31) $$\begin{array}{c} B\left[\tau \right]=\overset{\tau -1}{\underset{k=t_{o}(\tau )}{⋃}}B\left(k\right). \end{array}$$

In addition to the gene-insertion and mapping operation, the centroid repair *R* in CBAR is revised to further increase the performance of CBAR. The resulting CBAR evolves (as explained in Section 3.3.5) an initial population derived from the ID-mapped genotypes in *B*[*τ*].

Let *S* be the computing system used to solve the *ϕ*_*i*_^2^ subproblem, 0 ≤ *i* ≤ *L*, where *L* is a given value. One attribute of any resource in the environment is the ID of task that utilizes it. Thus, in system *S*, IDs are part of the information about (a) resources, (b) genotypes used in the evolutionary process of CBAR to solve the subproblem, (c) and task-precedence network of tasks in the environment. The insertion of new genes into each genotype in *G*(*k*) mentioned above is accompanied by the insertion of new nodes, with IDs of the new tasks, into the precedence network of tasks. The mapping of IDs in each gene-inserted genotype in *A*(*k*) mentioned above is accompanied by the mapping of IDs in the node-inserted precedence network of tasks and in the resources utilized by these tasks. When all IDs in system *S* are transformed by the mapping, system *S* is referred to as being in a mapped mode.

Under system *S* in mapped mode, in each cycle of the evolutionary process of CBAR, the ID-mapped genotypes in the *B*[*τ*]-derived initial population are inputted to the Schedule Formation method. This method then uses the mapped tasks IDs in resources and the precedence network of tasks in the (*t* − 1)th snapshot of the environment, to obtain schedules with mapped IDs. At the end of the evolutionary process, evolved genotypes have corresponding schedules, with mapped IDs, as solutions to *ϕ*_*τ*_^2^. Let *B*(*τ*) be the set of evolved genotypes which correspond to the nondominated solutions (schedules) to *ϕ*_*τ*_^2^.

Now, copies of the ID-mapped genotypes in *B*(*τ*) and the node-inserted ID-mapped task precedence network are made. Then IDs in each of the copies are unmapped. The ID-unmapped genotypes in *B*(*τ*) are processed in the Schedule Formation method that utilizes the ID-unmapped node-inserted task precedence network. Thereby, solutions to subproblem *ϕ*_*τ*_^2^ are produced. This production is referred to as* solution production*.

The copying and the subsequent unmapping of the ID-mapped genotypes are only performed when required, for example, for a decision maker, to view the solutions (schedules) to subproblem *ϕ*_*τ*_^2^. However, the evolved ID-mapped genotypes in *B*(*τ*) are utilized for finding solutions to problems set in the snapshots taken after the *τ*th SOSA of the environment. The gene-insertion, mapping/unmapping, initial population of gene-inserted genotypes, and the revision of the centroid repair *R* are fully described in Sections 3.4.1 to 3.4.4 below, respectively.

Suppose that no new tasks appear from the (*τ* + 1)th to the *σ*th SOSA of the environment, where *τ* < *σ* < *υ* and that the second batch of new tasks occurs at the *υ*th SOSA of the environment. Recall from the above discussion that gene insertion is performed only due to the occurrence of new tasks. Thus, there is no need to insert genes to each genotype in the populations *B*(*τ* + 1) to *B*(*σ* − 1) which correspond to the nondominated sets of solutions to the *ϕ*_*τ*+1_^2^ to *ϕ*_*σ*−1_^2^, respectively. The non-insertion of genes is depicted in Figure 7 as no arrows being pointed towards the SOSAs. Now, although the population *B*(*τ*) of genotypes that correspond to the nondominated solutions to *ϕ*_*τ*_^2^ is evolved from the initial population derived from the gene-inserted genotype populations in (31), genotypes in *B*(*τ*) had not undergone gene-insertion process. Thus, there is no downward-pointing arrow at *τ*th SOSA in the figure.

To solve the subproblem *ϕ*_*σ*_^2^, first, an initial population is formed using the populations in

(32) $$\begin{array}{c} B\left[\sigma \right]=\overset{\sigma -1}{\underset{k=t_{o}(\sigma )}{⋃}}B\left(k\right), \end{array}$$

where *t*_*o*_(*σ*) is defined in (28); *B*(*k*) could be a population of evolved ID-mapped gene-inserted genotypes—if *t*_*o*_(*σ*) ≤ *k* < *τ*—or a population of evolved ID-mapped uninserted genotypes—if *τ* < *k* < *σ*. Note that the insertion or noninsertion is depicted in Figure 7. Further, for *t*_*o*_(*σ*) ≤ *k* < *σ*, *B*(*k*) is the population of genotypes that correspond to nondominated solutions (schedules) to the *ϕ*_*k*_^2^ subproblem. Furthermore, *B*[*σ*] is a mix of populations of inserted and uninserted genotypes. Next, the initial population derived from *B*[*σ*] is evolved by CBAR to obtain a population *E*(*σ*) of evolved genotypes. Let *B*(*σ*)⊆*E*(*σ*) be a set of evolved genotypes that if fed to the solution production yield all the nondominated solutions to the subproblem *ϕ*_*σ*_^2^. Note that system *S* is at mapped mode during this evolutionary process.

Let us now consider the *υ*th SOSA of the environment where the second batch of new tasks occurs. Let *B*[*υ*] be a set of populations *B*(*k*), where *t*_*o*_(*υ*) ≤ *k* < *υ* and each could contain either ID-mapped gene-inserted genotypes or ID-mapped gene uninserted genotypes, a composition similar to that of (32). To solve subproblem *ϕ*_*υ*_^2^, first, the ID-mapped genotypes in *B*[*υ*] are unmapped to obtain a collection *D*[*υ*] of populations *D*(*k*), *t*_*o*_(*υ*) ≤ *k* < *υ*, of ID-unmapped genotypes. ID unmapping is also applied to task IDs in resources and in the precedence network of tasks in the (*υ* − 1)th snapshot of the environment. Thus, system *S* is restored to unmapped mode. Second, new genes that correspond to the second batch of new tasks are inserted into the unmapped genotypes in *D*[*υ*] to obtain a collection *E*[*υ*] of populations *E*(*k*), *t*_*o*_(*υ*) ≤ *k* < *υ* of ID-unmapped gene-inserted genotypes. This second batch of gene insertion is depicted in Figure 7 as upward-pointing arrows. New nodes, which correspond to the new tasks, are placed into the ID-unmapped task-precedence network. Third, a different mapping function is applied to IDs in each genotype in *E*[*υ*] to obtain

(33) $$\begin{array}{c} F\left[\upsilon \right]=\overset{\upsilon -1}{\underset{k=t_{o}(\upsilon )}{⋃}}F\left(k\right), \end{array}$$

which is a collection of populations *F*(*k*), *t*_*o*_(*υ*) ≤ *k* < *υ*, of ID-unmapped gene-inserted then ID-remapped genotypes. This mapping is also applied to the task IDs in resources and in the precedence network of tasks in the (*υ* − 1)th snapshot of the environment. Thus, system *S* is restored to mapped mode once again. Fourth, the collection *F*[*υ*] of population is used to form an initial population which is then evolved by CBAR to obtain the population *C*(*υ*) of evolved genotypes which has mapped IDs. Note again that system *S* is in mapped mode during the evolution of the initial population. Fifth, when required, the evolved genotypes in *C*(*υ*) are used in the solution production to obtain the non-dominated solutions (schedules) to the *ϕ*_*υ*_^2^ subproblem. The successive processes of (a) restoring system *S* to unmapped mode; (b) insertion of new genes that correspond to new tasks; and (c) reverting *S* to mapped mode, are repeated every time a batch of new tasks appear in the environment after the first batch.

Based on the discussions until this point, system *S* is always in mapped mode when CBAR evolves initial populations to determine solutions to subproblems set at or after the snapshot of the environment where the first batch of new tasks are found. In addition, the formation of the initial population uses the genotype populations that correspond to sets of nondominated solutions.

CBAR that undergoes all of the above-mentioned innovations is labeled as McBAR. In summary, McBAR is comprised of the following subalgorithms used to solve subproblem *ϕ*_*t*_^2^ set in the *t*th SOSA of the environment:

1. gene insertion,
2. ID mapping operation *F*,
3. minimal repair *M* of centroid,
4. initial population defined in (34) below,
5. NSGA-II (explained in Section 3.3.5) used to evolve the initial population,
6. maintenance of system *S* at mapped mode,
7. preservation of ongoing and finished tasks in a chosen schedule (described in Section 3.3.6),
8. SSGS to form *G*_0_(0) and Rnd(*t*) in  (26).

Items (1) to (4) are fully discussed in Sections 3.4.1 to 3.4.3, respectively. The algorithm of McBAR is similar to that of CBAR (described in Section 3.3.5), except that items (1) to (4) are applied to form an initial population and that system *S* is maintained at mapped mode, that is, item (6). Based on the definition of performance in Section 2.3, McBAR was demonstrated in [14] to perform better than CBAR when the environment undergoes an increase in total number of tasks.

#### 3.4.1. Gene Insertion

**Figure 8:**
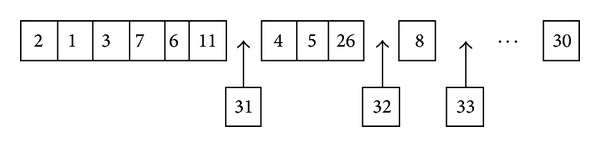
Sample gene insertion.

Suppose that new tasks appeared at the *τ*th SOSA of the environment and consider also a genotype *G* in a collection *G*[*τ*] (e.g., defined in (30) to (33)) of genotype populations. The new gene that corresponds to a new task is inserted to the right of and as near as possible to the gene, currently in *G*, that corresponds to the immediate predecessor of the new task. This gene insertion abides by the gene ordering rule (mentioned in Section 3.1.5) of genes in any genotype. It is performed for each of the new tasks. The complete insertion process of the new genes is applied to every genotype in *G*[*τ*]. Sample gene insertion is illustrated in Figure 8 where genes are represented by boxed numbers. Genes/IDs 31 to 33 correspond to the new tasks and the rest of IDs to those of original tasks (the only tasks found in the original state of the environment). The gene arrangement in this figure abides by the gene ordering rule that uses the task-precedence network in Figure 4 with circles that represent tasks 34 to 40 replaced with arcs.

Empirical investigations in [14] showed gene insertion to degrade the performance (as defined in Section 2.3) of McBAR. In the research of [16, 19–21], gene insertion was shown to be beneficial for searching for high quality solutions to some problems. However, there is no centroid (as defined in (23)) utilized in their strategies, such that the benefit does not necessarily apply to the performance of McBAR.

Let us describe a possible cause of the degradation in the performance of McBAR. It is possible that new tasks in the environment cannot be anticipated; this could happen in practice. It is then sensible to label original tasks in the environment with IDs from one to *N*, the number of original tasks. In this set-up, new genes that correspond to the new tasks must have task IDs (e.g., 31 to 33 in Figure 8) greater than *N*. Now, as exemplified in Figure 8, the insertion of the new genes brings about a large discontinuity of task ID values along a gene-inserted genotype. As demonstrated in [14], this large discontinuity is a factor in the degradation of the performance of McBAR; that is, the large ID discontinuity is a gene insertion side effect.

#### 3.4.2. Resolution of Effects

Before proceeding to discuss the resolution of the gene insertion side effect, let us define the precedence order of a task. This order is the maximum number of directed links that connect the task to the start of a given task precedence network following the reversed direction of the links. For instance, task 16 in Figure 4 is of seventh precedence order.

The insertion side effect was resolved in [14] by mapping IDs of tasks, that belong to a similar precedence order, to unique values that are as close to each other as possible. Let the function *F*(*I*_*d*_) represent the ID-mapping operation, where *I*_*d*_ is the ID to be mapped. For example, second precedence ordered tasks in Figure 4 such as 4, 6, 7, 8, 9, 10, 12, 14, and 38 are mapped to themselves, except task 38 which is mapped to 15. As empirically demonstrated in [14], the mapping *F* reduces, with high likelihood, the abrupt change of IDs along a gene-inserted genotype.

Note that although a task ID may be transformed, the task that it represents remains the same. For example, the starting time of and the type of resource utilized by the task are not affected by *F*, except the task ID in the utilized resource. Further, aside from mapping task IDs in genotypes and resources, node IDs in an associated task precedence network are also mapped by *F*.

#### 3.4.3. Initial Population for a Current Static Subproblem

The subproblem *ϕ*_0_^2^ set in the original state of the environment is solved by McBAR by evolving an initial population composed of *N* SSGS-generated genotypes. The initial population used to determine the solutions to subproblem *ϕ*_*t*_^2^, where *t* > 0, is formed as follows. Suppose that new tasks first appear at the *τ*th SOSA of the environment. Let *B*[*t*] be a collection of genotype populations *B*(*k*), *t*_*o*_(*t*) ≤ *k* < *t*, where *t* ≥ *τ* and *t*_*o*_(*t*) is defined in (28). *B*(*k*) could be a population of gene-inserted ID-mapped genotypes (e.g., *B*(*k*)s in (31) or *F*(*k*)s in (33)) or a population of uninserted ID-mapped genotypes (e.g., *B*(*k*)s in (32)). As mentioned above, genotypes in *B*(*k*) correspond to the nondominated solutions to the *ϕ*_*k*_^2^ subproblem. The collection *B*[*t*] is used to form an initial population,

(34) $$\begin{array}{c} M_{0}\left(t\right)=C\left(t\right)\cup \text{Rnd}\left(t\right)\cup \text{Mchs}\left(t-1\right), \end{array}$$

where

(1) *C*(*t*) is a set of centroids,
  (35) $$\begin{array}{c} C\left(t\right)=\overset{t-1}{\underset{k=t_{o}(t)}{⋃}}R\left(k\right); \end{array}$$
(2) *R*(*k*) is the *M*-repaired (described below) centroid of *B*(*k*) ∈ *B*[*t*];
(3) Rnd(*t*) is the set of genotypes generated through SSGS which utilizes the ID-mapped and could be node-inserted precedence network of tasks in the (*t* − 1)th snapshot of the environment. |Rnd(*t*)| = *N* − |*C*(*t*)| − 1. Being produced through SSGS (which endows stochasticity to its produced genotypes), the elements of Rnd(*t*) are referred to as random immigrants. As noted in Section 2.2, memory-based EA approaches need to diversify the solutions at some stages of their evolutionary cycles. In McBAR, this diversification is implemented through the random immigrants, thereby justifying the inclusion of Rnd(*t*) in (34);
(4) *N* is a fixed size of the initial population;
(5) Mchs(*t* − 1) is the ID-mapped chosen genotype in *B*(*t* − 1) which corresponds to the chosen schedule from the population *P*(*t* − 1) of nondominated solutions to the *ϕ*_*t*−1_^2^ subproblem.

Based on the enumerated definitions above, all components of the initial population *M*_0_(*t*) have genotypes with mapped IDs. This initial population is then evolved (as explained in Section 3.3.5) by McBAR to determine an ID-mapped evolved population. When necessary, this evolved population is fed to the solution production method to obtain a set *P*(*t*) of schedules as solutions to the *ϕ*_*t*_^2^ problem.

#### 3.4.4. Centroid Repair

The centroid components of (35) could be task-precedence infeasible based on the ID-mapped node-inserted precedence network of tasks in the (*t* − 1)th snapshot of the environment. McBAR repairs task-precedence infeasible centroids using a function *M* different to *R* of CBAR. The *M* centroid repair function perturbs the centroids as little as possible such that, intuitively, their being centroids will diminish least.

Recall from Section 3.3.1 that the ID *ρ* of a centroid *C* is allowed to be appended to a genotype *R* if it satisfies the appending rule. Otherwise, the function *R* of CBAR randomly picks a different ID *ρ*′, that satisfies the appending rule, from *R* ^*c*^ = *C* − *R* and then appends this to *R*, where *R* ^*c*^ is the complementary genotype of *R*. Now, the function *M* of McBAR differs from *R* in the following. Instead of randomly picking an element of *R* ^*c*^, the element of *R* ^*c*^ that is nearest in value to *ρ*, and satisfies the appending rule, is the one appended to *R*. If two IDs from *R* ^*c*^ are equidistant to *ρ* and satisfy the appending rule, one of these is randomly picked for appending to *R*. The appending rule uses the node-inserted ID-mapped precedence network of tasks in the (*t* − 1)th snapshot of the environment if *t* in (34) is greater than or equal to *τ* which is the SOSA of the environment at which new tasks appear for the first time. If <*τ*, the appending rule uses the precedence network of original tasks.

As an example, consider the centroid *C* = 〈14,1, 2,7, 9,3,…〉; its first gene/ID 14; the current *R* = *∅*; and *R* ^*c*^ = *C*. As mentioned in Section 3.3.1, ID 14 does not satisfy the appending rule. However, the ID 3 from *R* ^*c*^ satisfies the appending rule and is nearest to 14. Consequently, *R* = 〈3〉 and *R* ^*c*^ becomes 〈14,1, 2,7, 9,…〉. The ID 1 of *C* is considered for appending to *R* next. It satisfies the appending rule such that *R* = 〈3,1〉. The ID 2 of *C* is considered next and also satisfies the appending rule, such that *R* = 〈3,1, 2〉.

Let the first three genes of *C* form the vector *V* = 〈14,1, 2〉 and the last *R* be treated as vector. The last *R*, produced through the *M* function, has a distance of 11 to *V*, while *R* = 〈1,14,2〉, produced (as mentioned in Section 3.3.1) through the *R* function, has a distance of 18.38 to *V*. This result supports the lesser perturbation effected by *M* than by *R* on repairing centroids.

#### 3.4.5. Using the Median

The centroid expressed in (23) is computed using the mean which is one type of tendency in statistics [52]. It is of interest to determine the performance of McBAR when the centroid is computed using a median, in which case the centroid is renamed as medoid and McBAR as* MedianBAR*. The *i*th gene of a medoid is

(36) $$\begin{array}{c} M_{i}\left(t\right)=\{\begin{array}{cc} s_{i}^{M+1}\left(t\right) & N\;\text{is}\;\text{odd}\;\\ \left\lfloor \frac{s_{i}^{M}\left(t\right)+s_{i}^{M+1}\left(t\right)}{2}\right\rfloor & N\;\text{is}\;\text{even}, \end{array} \end{array}$$

where *s*_*i*_^*k*^ is an element of the ordered set *S*(*t*) = 〈*s*_1_^*j*^(*t*), *s*_2_^*j*^(*t*),…, *s*_*N*_^*j*^(*t*)〉; *S*(*t*) = sort(*X*(*t*)); *X*(*t*) = {*x*_1_^*j*^(*t*), *x*_2_^*j*^(*t*),…, *x*_*N*_^*j*^(*t*)}; sort is a sorting operation; *x*_*i*_^*j*^(*t*) is the task ID in the *i*th gene of the *j*th genotype in a population of size *N*; and *M* = ⌊*N*/2⌋.

### 3.5. Other Techniques

This section provides information on techniques, other than CBAR, McBAR, and MedianBAR, that are utilized to legitimize some components of McBAR, that is, to achieve goal 1 in the Introduction.

#### 3.5.1. EDA on Φ^2^ Problem

The EDA algorithm described in Section 2.5 is innovated, in order to solve problem Φ^2^, and consequently labeled as EDA/Φ^2^. Recall from Section 3.1.1 that subproblem *ϕ*_0_^2^ is set in the original state of a dynamic environment. With *t* = 0, EDA/Φ^2^ determines a set of solutions to *ϕ*_*t*_^2^ through the following steps, starting with EDA cycle *i* = 0.

(1) The probability matrix in (6) is relabeled as Pm_*i*_(*t*) to indicate the *t*th SOSA of the environment that sets the problem which it is being used to solve. It is set to have equal entries of 1/*Nt*; that is, Pm_*i*_(*t*) = [1/*Nt*], where *Nt* is the total number of tasks at the *t*th snapshot of the environment. This step is the equivalent of EDA/Φ^2^ to step 1 in Algorithm 2.
(2) The probability matrix Pm_*i*_(*t*) is then sampled, following the sampling scheme in Section 2.5, to form a population *G*_*i*_(*t*) of *N* genotypes. This step is related to step 2 in Algorithm 2.
(3) From *G*_*i*_(*t*), a set *S*_*i*_(*t*) of schedules is formed through the schedule formation scheme described in Section 3.3.4.
(4) Costs and makespans of schedules in *S*_*i*_(*t*) are utilized in NSGA-II's fast nondominated sorting to obtain each schedule's Pareto rank and crowding distance [40].
(5) *N*_sel_ = ⌈*ρN*⌉ genotypes are selected from *G*_*i*_(*t*), where ⌈·⌉ is a round-up operator; 0 < *ρ* ≤ 1; and *ρ* is a predefined constant referred to as percent of population. This selection is performed following the scheme described in Section 2.4 and is based on the Pareto rank and crowding distance of schedules in *S*_*i*_(*t*). This step is related to step 3 in Algorithm 2.
(6) These *N*_sel_ selected genotypes are included to *N* − *N*_sel_ SSGS-generated genotypes to form a new population *G*_*i*+1_(*t*) of size *N*. Note that, as explained in Section 3.3.2, SSGS-generated genotypes add diversity to the population in which they are included. The inclusion of the SSGS-generated genotypes is intended to remedy the loss of diversity of the population produced by EDA as the evolutionary cycles of EDA progress [44].
(7) A new probability matrix Pm_*i*+1_(*t*) is estimated from the new population *G*_*i*+1_(*t*). Following [43], the probability matrix to be used in the next step is
  (37) $$\begin{array}{c} \text{P}{\text{m}}_{i+1}\left(t\right)⟵\lambda \text{P}{\text{m}}_{i+1}\left(t\right)+\left(1-\lambda \right)\text{P}{\text{m}}_{i}\left(t\right), \end{array}$$
  where 0 < *λ* ≤ 1 is a chosen value and is referred to as a learning rate. This equation allows the information, embedded in Pm_*i*_(*t*), from the last cycle to be carried over (learned) to the next EDA cycle. This step is related to step 5 in Algorithm 2.
(8) Steps  2 to 7 are repeated for a fixed number *N*cyc of generations, except at the last generation where only steps 2 and 3 are executed successively. The generation index *i* is incremented at every end of the generations. At the last generation, where *i* = *N*cyc, the evolved population *G*_*N*cyc_(*t*) is utilized in the schedule formation scheme in Section 3.3.4 to form a set *S*_*N*cyc_(*t*) of schedules as solutions to the subproblem *ϕ*_*t*_^2^. Let *S*_*N*cyc_(*t*) and *G*_*N*cyc_(*t*) be relabeled as *P*(*t*) and *G*(*t*), respectively.

Steps  6 and 1 are revised to determine solutions to subproblem *ϕ*_*t*_^2^ with *t* > 0. Step  6 is replaced as follows. The *N*_sel_ selected genotypes are included with *N* − *N*_sel_ − 1 SSGS-generated genotypes and with a chosen genotype to complete a population *G*_*i*+1_(*t*) of size *N*. The chosen genotype corresponds to the chosen schedule randomly picked from the set Pnds(*t* − 1) of nondominated solutions in *P*(*t* − 1). The finished and ongoing tasks in the chosen schedule are preserved (as explained in Section 3.3.6) in the evolved schedules as solutions to the subproblem *ϕ*_*t*_^2^.

When there is no increase in the total number of tasks at the *t*th SOSA of the environment, *t* > 0, step 1 is replaced as follows. Let

(38) $$\begin{array}{c} G\left[t\right]=\overset{\tau -1}{\underset{k=t_{o}(t)}{⋃}}\text{Gnds}\left(k\right), \end{array}$$

where *t*_*o*_(*t*) is defined in (28) and Gnds(*k*) is a set of genotypes that correspond to all nondominated solutions in the set *P*(*k*) of solutions to subproblem *ϕ*_*k*_^2^. From *G*[*t*], the probability matrix Pm_*i*_(*t*) is estimated. In this way, solutions (e.g., Gnds(*k*)) to subproblems set in past snapshots are utilized to search for solutions to the current (*t*th) snapshot of the environment.

If there is an increase in the total number of tasks, say at the *t*_*n*_th SOSA of the environment, *t*_*n*_ > 0, step 1 is replaced as follows. New genes that correspond to new tasks which appear at the *t*_*n*_th SOSA are inserted into all genotypes in *G*[*t*] of (38) following the scheme in Section 3.4.1. From the gene-inserted [*t*], the *Nt* × *Nt* probability matrix Pm_*i*_(*t*) is estimated, where *Nt* is the total number of tasks at the *t*_*n*_th SOSA.

The algorithm of EDA/Φ^2^ above implies that any genotype it processed has a fixed length during its evolutionary process. Thus, following the discussion in Section 3.3.7, the gene insertion is a legitimate step.

#### 3.5.2. Techniques

As mentioned in Introduction, goal 1 in this paper is to legitimize some subalgorithms of McBAR. These subalgorithms are items 2 to 5 of those enumerated in Section 3.4. The legitimization is based on the idea that if technique *A* differs from technique *B* in regard to some components, and if, in general, *A* performs better than *B* in solving a given set of problems, then the components of *A distinct* from *B* are legitimate for *A* to solve this set of problems. Consider a sample case of the legitimization. Both McBAR and EDA/Φ^2^ (described in Section 3.5.1) individually solve some instances of problem Φ^2^. Note that McBAR uses EA operators while EDA/Φ^2^ uses distribution sampling to produce offspring. If in solving most of these instances, McBAR performs better than EDA/Φ^2^, then the EA operators utilized in McBAR are legitimate components of McBAR to solve these instances. To accomplish the legitimization goal, a set *T* of techniques is formed comprised of McBAR and other techniques utilized to legitimize some subalgorithms of McBAR. These other techniques are as follows.

(1) Centroid-based adaptation with minimal repair (CBAM) differs from GIBAR (described in Section 3.4) only in using *M* instead of *R* to repair centroids. Based on the descriptions in Sections 3.3 and 3.4 of the subalgorithms of CBAR and McBAR, respectively, GIBAR differs from McBAR in using *R*, in the mapping *F* of task IDs in genotypes, and in the maintenance of system *S* at mapped mode. Considering that CBAM differs from GIBAR by using *M* which is also used by McBAR, then it differs from McBAR in the mapping *F* and in the maintenance of system *S* at mapped mode. Implied in Section 3.4, the application of the mapping *F* causes the maintenance of the system at mapped mode. Considering this catalysis, the fundamental difference between CBAM and McBAR is only in regard to *F*. This difference will be used as the basis of legitimizing the *F* in McBAR.

(2) Mapping of task IDs for centroid-based adaptation with stochastic repair (McBAS) differs from McBAR only in using random centroid repair *R* instead of the minimal centroid repair *M*. This difference will be used as the basis of legitimizing the *M* in McBAR.

(3) Mapping of task IDs for centroid-based adaptation (McBA) differs from McBAR in randomly selecting genotypes from the set Gnds(*t* − 1) of genotypes that correspond to the nondominated solutions to the last subproblem *ϕ*_*t*−1_^2^, instead of generating genotypes through SSGS, to form the Rnd(*t*) component of the initial population in (34), where *t* corresponds to the current subproblem *ϕ*_*t*_^2^ being solved. This difference will be used as the basis of legitimizing the random immigrant component (SSGS-generated genotypes) in McBAR.

(4) Mapping of task IDs for centroid-based adaptation with medoids (MedianBAR) differs from McBAR only in using the median (defined in (36)) instead of the mean to compute for the centroid *R*(*k*) in (34). This difference will be used as the basis of legitimizing the process of taking the mean in McBAR.

(5) EDA/Φ^2^ is defined in Section 3.5.1 and differs from McBAR in its use of sampling and the estimation of a probability matrix (described in Section 2.5), instead of using mutation and crossover operators, to form the next generation offspring in its evolutionary process. Note that, based on Section 3.5.1, EDA/Φ^2^ does not apply the mapping *F* function, maintain system *S* in mapped mode, and repair the centroid using *M*. Based on Section 3.4, these subalgorithms of McBAR are devised due to the use of centroids as memory in McBAR. On the other hand, EDA/Φ^2^ uses the probability matrix as memory, based on the revised step 1 in Section 3.5.1. Thus, McBAR fundamentally differs from EDA/Φ^2^ in the use of EA operators and the centroid. These differences will be used as the basis of legitimizing the use of the EA operators and the centroid in McBAR.

(6) NDS of last population (NDLPOP) differs from GIBAR in randomly selecting genotypes, from the above-mentioned set of genotypes Gnds(*t* − 1), to form the *C*(*t*) component of the initial population in (26). Note that the resulting *C*(*t*) is no longer a set of centroids but rather of genotypes of Gnds(*t* − 1). Thus, NDLPOP differs from McBAR in being an explicit memory-based approach. This difference will be used as the basis of legitimizing the implicit memory-based approach of McBAR.

It is also of interest to compare the performance of McBAR and the previously enumerated techniques to those of the following techniques.

(7) Random immigrants (RI) creates an initial population for NSGA-II to evolve through SSGS. The length of each genotype in this initial population is equal to the total number of tasks right after the environmental change of state. This technique differs from other techniques in *T* in applying no rule in creating its initial population, except the rules followed by SSGS.

(8) Gene-inserting CBAR (GIBAR) is described in Section 3.4.

Techniques that apply the mapping function *F*, such as McBAS, McBA, McBAR, and MedianBAR, are classified as variants (of McBAR) and the rest of techniques in *T* as nonvariants.

The parametric values of EDA/Φ^2^ and other techniques in *T* are listed in Tables 6(b) and 6(a), respectively. These values enable each of the techniques to yield high quality solutions to a significant problem instance of Φ^2^ and are determined through an approach, described in our other work [53, 54]. In our preliminary investigations, the performances of the techniques in *T* other than EDA/Φ^2^ are high with evolutionary processes that have selection rate of 0.5. Further, the performances of all techniques in *T* are found to stabilize before the 300th generation of the evolutionary processes in these techniques. Thus, the evolutionary processes in the techniques are terminated at this value. The approach to determine the tabulated parametric values of EDA/Φ^2^ is explained in Section 4.1.

## 4. Results and Discussions

Let us explore the results of solving problem instances of Φ^2^, whose labels are listed in Table 5, by each technique in *T* (defined in Section 3.5.2). Section 4.1 presents the results of determining some performance-enhancing parametric values of EDA/Φ^2^. Note that some EA-parametric values of techniques in *T* other than EDA/Φ^2^ were determined in our other work [53, 54].  Section 4.2 provides information on the relative performances of the techniques with respect to the parameter *δ* of the amount of change in duration of tasks in environments that set the instances. Based on these relative performances, the subalgorithms of McBAR are legitimized in Section 4.3.  Section 4.4 presents the relative performances of the techniques with respect to the type of changes that occur in the environments.  Section 4.5 discusses the dynamics of the relative performances with respect to the SOSA of the environments. For brevity, let the environments that set the instances of Φ^2^ whose labels are listed in Table 5 be referred in this section as the environments.

### 4.1. EDA/Φ^2^ Parametric Values

**Figure 9:**
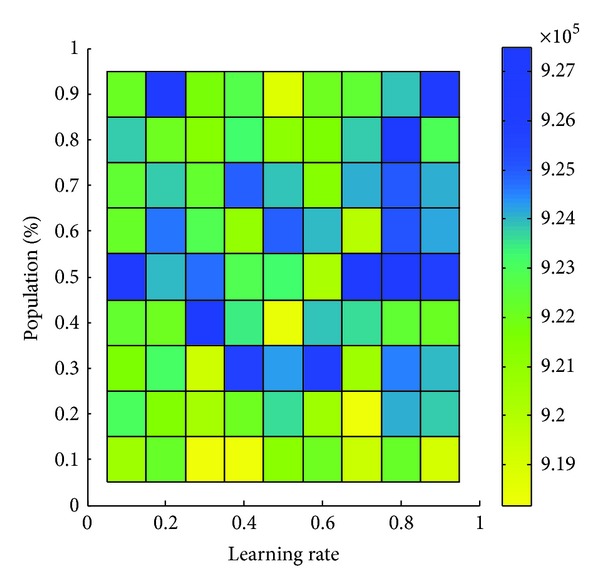
Performance of EDA/Φ^2^ at various values of its parameters.

The values of the learning rate *λ* (defined in item 7) and the percentage *ρ* (defined in Item 5) at which the performance of EDA/Φ^2^ is boosted are determined as follows. The parameters *λ* and *ρ* are restricted to take values in the range 0.1 to 0.9 with a 0.1 interval. For each pair of permissible *λ* and *ρ* values, EDA/Φ^2^ solves a significant instance of Φ^2^ defined in our other work [53, 54]. Then a performance as defined in terms of hypervolume [55] is computed from the solutions obtained by EDA/Φ^2^ that uses the pair in solving the instance. The performances that correspond to all of the pairs are plotted in Figure 9. This figure illustrates that the performances are generally high at *λ* greater than 0.6 and *ρ* greater than 0.4. Among the pairs, EDA/Φ^2^ performs best at *λ* and *ρ* each being 0.8.

### 4.2. Relative Performance with respect to Task Duration Change

Let us now discuss the influence of task duration changes on the average **E**_*t*_^*δ*^[·] (defined in (19)) relative performances of techniques in *T* in solving problem instances of Φ^2^. For brevity, let the average **E**_*t*_^*δ*^[·] be referred simply as performance in this and the next subsection.

Table 7(a) presents the performances **E**_*t*_^*δ*^[dSC(*A*, *B*)] of technique *A* over technique *B* which are found in the first column and first row of this table, respectively. These performances are derived from the solutions determined by the techniques in solving problem instances of Φ^2^ that have task duration changes parameterized by *δ* = 3.0 through (11). Referring to the second row of this table, **E**_3.0_^*δ*^[dSC(GIBAR, *T*)] > 0 only when technique *T* is either NDLPOP, RI, or EDA/Φ^2^. Thus, GIBAR performs better than NDLPOP, RI, and EDA/Φ^2^. Note that the degree of performance is based on the definition in Section 3.2.4. Succeeding rows in this table demonstrate that the performance of CBAM is inferior only to that of variants (defined in Section 3.5.2); the performance of NDLPOP is superior only to that of EDA/Φ^2^ and RI; the performance of RI is superior only to that of EDA/Φ^2^; the performance of EDA/Φ^2^ is inferior to all other techniques; the performance of McBA is inferior to that of other variants and superior to that of nonvariants; the performance of McBAR is inferior to that of MedianBAR only; the performance of McBAS is inferior to that of McBAR and MedianBAR only; and the performance of MedianBAR is superior to that of all other techniques. Note however that the degree of MedianBAR's superiority to McBAR is small, **E**_3.0_^*δ*^[dSC(McBAR, MedianBAR)] = 0.01, that is, near zero. Data in this table showed variants to be superior to nonvariants.

Table 7(b) presents the performances of technique *A* over technique *B* which are found at the first column and first row of this table, respectively. This average is determined by the techniques in solving problem instances of Φ^2^ that have task duration changes parameterized by *δ* = 6.0 through (11). Table 7(b) illustrates that the performances between the techniques are generally similar to those of Table 7(a), except that the performance of McBAR is superior to all other techniques, especially on MedianBAR, and that the performance of MedianBAR is inferior by a small degree to that of McBAR, **E**_6.0_^*δ*^[dSC(MedianBAR, McBAR)] = −0.01. In view of these findings and considering that Tables 7(a) and 7(b) correspond to *δ* = 3.0 and *δ* = 6.0, respectively, the performances of techniques in both tables are generally not affected by the two values of *δ*.

Let the rank of superiority in performance of a technique over other techniques in *T* be the number of these other techniques on both tables to which it is superior. The techniques in *T* arranged in descending order of superiority are McBAR, MedianBAR, McBAS, McBA, CBAM, GIBAR, NDLPOP, RI, and EDA/Φ^2^ whose performances are superior to 15, 15, 12, ten, eight, six, four, two, and zero techniques in the tables, respectively.

### 4.3. Legitimization

Let us now legitimize subalgorithms of McBAR using, the legitimization principle presented in Section 3.5.2, the above results and the differences of McBAR to other techniques described in Section 3.5.2. Note that item numbers cited in this subsection refer to items in the enumeration in Section 3.5.2. Based on the legitimization principle, the superiority of McBAR to CBAM (Item 1) legitimizes its use of mapping *F* since, as discussed in Section 3.5.2, it fundamentally differs from CBAM on this subalgorithm. Its superiority in performance to McBAS legitimizes its use of the minimum centroid repair *M* since it differs from McBAS on this mapping only. Its superiority in performance to McBA (Item 3) legitimizes its use of the random immigrant component *S*(*t*) in (34) since it differs from McBA by this component only. As noted in Section 3.3.2, random immigrants can diversify an initial population that includes them. Thus, the superiority of McBAR over McBA supports the finding in [31] on the relevance of diversification of population in some EA evolutionary processes. The approximately equal performance of McBAR to MedianBAR (Item 4) does not legitimize its use of the mean to compute the centroid through (23). The superiority in performance of McBAR to NDLPOP (Item 6) legitimizes its use of an implicit memory-based approach since it differs from NDLPOP on this type of approach. Its superiority in performance to EDA/Φ^2^ (Item 5) legitimizes its combined use of EA operators and centroids since it fundamentally differs from EDA/Φ^2^ on these components.

Table 8 lists the average number of CPU cycles, on identical computing machines, required by techniques in *T* to determine solutions to Φ^2^ problem instances whose labels are listed in Table 5. This average is taken over a similar domain as in the average **E**_*d*_^*δ*^[·] in (19). Based on the column with the heading *δ* = 3.0, nonvariants require lesser CPU cycles to determine the solutions than variants. Among the variants, MedianBAR requires the most number of CPU cycles to determine the solutions. This last result is expected since the computation of the median is more algorithmically complex than the computation of the mean based on (36) and (23), respectively. The relationships of the numbers of CPU cycles for the column heading *δ* = 3.0 are also true for the column heading *δ* = 6.0. Thus, the relationships are not affected by the two values of *δ*.

McBAR requires less computational expense than MedianBAR to determine the solutions to all problem instances in Table 5; it has superior (as illustrated in Table 7(b)) performance to MedianBAR in solving problem instances in Table 5 whose task duration changes are parameterized by *δ* = 6.0 and it has superior (as illustrated in both Tables 7(a) and 7(b)) performance to all techniques, other than itself and MedianBAR. Despite the inferior (as illustrated in Table 7(a)) performance of McBAR to MedianBAR in solving problem instances in Table 5 whose task duration changes are parameterized by *δ* = 3.0, these findings show McBAR to be the most versatile technique among all the techniques in *T*.

### 4.4. Relative Performance with respect to Type of Changes

Let us now explore the influence of the type of changes in the environment on the average **E**_*t*_^*τ*^[·] (defined in (20)) relative performances of techniques in *T* in solving problem instances of Φ^2^ set in the environment. For brevity, let the average **E**_*t*_^*τ*^[·] be referred to simply as performance in this subsection.

Tables 9 to 11 present the performances **E**_*t*_^*τ*^[dSC(*A*, *B*)] where *A* is the technique name under the column heading “technique” and *B* is the technique name in the table heading. Further, the integer under the column heading “type” is the change type label *t* enumerated in Table 2. In the first row of Table 9, **E**_0_^*τ*^[dSC(CBAM, McBA)] = −0.25, **E**_0_^*τ*^[dSC(CBAM, McBAR)] = −0.23, **E**_0_^*τ*^[dSC(CBAM, McBAS)] = −0.21, and **E**_0_^*τ*^[dSC(CBAM, MedianBAR)] = −0.25. Thus, GIBAR performs more poorly than all variants when the environment changes in task duration only (type 0 in Table 2). CBAM, NDLPOP, and RI also perform worse than variants at change type 0 based, respectively, on the second to the fourth rows of the type 0 group of rows.

The performance of the nonvariants, in the type 5 group of rows in Table 10, is inferior to that of the variants when the change in the environment is of type 5 (simultaneous changes in task duration and number). The degree of inferiority is of an approximately similar degree as to that which is found when the change in the environment is of type 0. The performance of nonvariants, in type 1 group of rows in Table 9, is inferior to that of variants when the change in the environment is of type 1 (change in resource availability). This inferiority is of higher degree when the change in the environment is of type 0; for example, **E**_0_^*τ*^[dSC(CBAM, McBAS)] < **E**_1_^*τ*^[dSC(CBAM, McBAS)].

The type 2 group of rows in Table 9, type 3 group of rows in Table 10, and type 6 group of rows in Table 11 demonstrate that the performance of the nonvariants is inferior to that of the variants when the changes in the environment are of types 2, 3, and 6, respectively. This inferiority is greater when the change in the environment is of type 1. Note that change types 2, 3, and 6, based on Table 2, involve change in the total number of tasks. The above results manifest the superiority of variants over nonvariants when the changes in the environment involve change in total number of tasks, that is, change types 2, 3, 5, and 6. However, the variants in the type 4 group of rows in Table 9 perform less well than CBAM, NDLPOP, and RI when the change in the environment is of type 4 which, according to Table 2, denotes simultaneous changes in task duration and resource availability, for example, a type of changes that does not involve change in the total number of tasks. Tables 9 to 11 illustrate the inferior performance of EDA/Φ^2^ to all other techniques in *T* with any type of changes in the environment. Further, they manifest the approximately similar performances of McBAR and MedianBAR compared to other techniques in *T*.

### 4.5. Dynamic Performance

Let us now investigate the influence of the dynamics of the environment on the average **E**_*j*,*k*_^*σ*^[·] (defined in (21)) relative performances of techniques in *T* in solving problem instances of Φ^2^ set in the environment. As mentioned above, the performance of EDA/Φ^2^ on average is inferior to all other techniques in *T* in solving the problem instances. Further, McBAR and MedianBAR have approximately similar performances, on another average, relative to other techniques in *T*. Not shown, the dynamics of performances, based on the **E**_*j*,*k*_^*σ*^[·] average, of GIBAR and CBAM relative to other techniques in *T* are not significantly different. By these observations EDA/Φ^2^, GIBAR, and MedianBAR are excluded from the following discussions. The included techniques are NDLPOP, RI, McBA, McBAR, McBAS, and CBAM.

For brevity, let the average **E**_*j*,*k*_^*σ*^[dSC(*A*, *B*)] be referred to simply as the performance in the remaining portion of this section. Before further discussion, let us describe a certain figure format crucial in presenting the dynamics of the performances of the included techniques.

#### 4.5.1. Figure Arrangement

**Figure 10:**
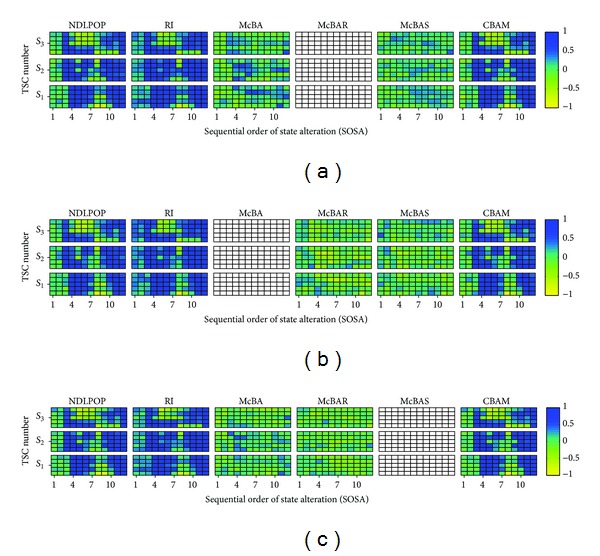
Dynamics of **E**_*j*,*k*_^*σ*^[·] with *δ* = 3.0 with (a) McBAR, (b) McBA, and (c) McBAS as basis techniques.

**Figure 11:**
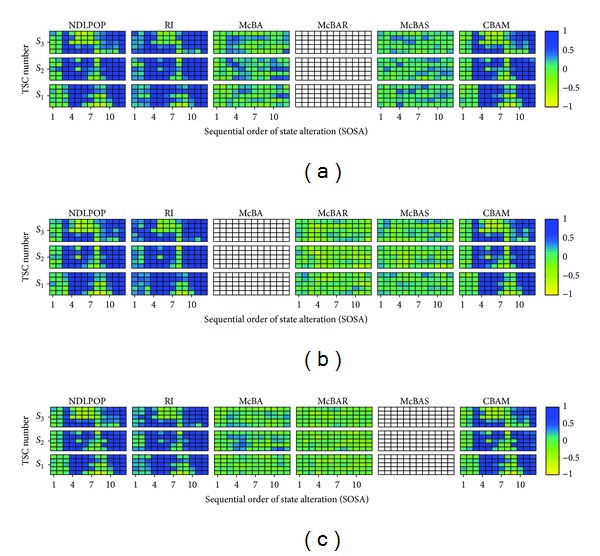
Dynamics of **E**_*j*,*k*_^*σ*^[·] with *δ* = 6.0 with (a) McBAR, (b) McBA, and (c) McBAS as basis techniques.

The average **E**_*j*,*k*_^*σ*^[dSC(*T*, *S*)] is illustrated in Figures 10 and 11 for some combinations of its indices *j* and *k*, related as *j*th subproblem of instance *k* listed in Table 5, where 0 ≤ *j* ≤ *L* and *L* is defined in Section 3.2.1. In Figure 10(a), the heading of the block of white colored small squares is the name of technique *T*, called basis technique (e.g., McBAR). Further, the headings over colored blocks are names of techniques denoted by *S*. For example, the middle block of Figure 10(a) under the heading McBAS corresponds to averages **E**_*j*,*k*_^*σ*^[dSC(McBAR, McBAS)], for various combinations of indices *j* and *k*. This technique-heading correspondence applies to all other subfigures of Figures 10 and 11.

In Figure 10(a), row of blocks at similar level as the label *S*_*i*_ at the left of this figure correspond to TSC *S*_*i*_ listed in Table 5, where 1 ≤ *i* ≤ 3. For example, the middle blocks correspond to *S*_2_. A colored block in the figure is denoted by the *S*_*i*_ label which is of similar level as this block and by the heading under which this block is found. For example, *S*_2_-RI block is the middle block under the RI column heading. Blocks at the same level as the *S*_*i*_ label also correspond to an ordered set *N*_*i*_^3.0^ of Φ^2^ instances listed in Table 5 where

(39) $$\begin{array}{c} N_{1}^{3.0}=\langle 1,7,13,19,25\rangle , \end{array}$$

(40) $$\begin{array}{c} N_{2}^{3.0}=\langle 2,8,14,20,26\rangle , \end{array}$$

(41) $$\begin{array}{c} N_{3}^{3.0}=\langle 3,9,15,21,27\rangle . \end{array}$$

For example, the middle blocks correspond to *N*_2_^3.0^. Inside a block at the same level as the *S*_*i*_ label, the rows from bottom to top correspond to elements of *N*_*i*_^3.0^, respectively. For example, the bottom to top rows of *S*_2_-RI block correspond to instances 2, 8, 14, 20, and 26, respectively.

The horizontal coordinate of each small square in each block corresponds to the *j*th SOSA of the environment that sets problem instance in *N*_*i*_^3.0^. Continuing the example, the fourth small square from the left on the third row of the *S*_2_-RI block corresponds to the fourth SOSA of the environment that sets instance 14 (the third element of *N*_2_^3.0^); that is, it corresponds to problem instance 14_4_^2^ (refer to Section 3.2.1 for notation). The colorbar at the furthest right of the figure maps colors in the squares to values. In the example, the color of the small square that corresponds to problem instance 14_4_^2^ represents **E**_4,14_^*σ*^[dSC(McBAR, McBAS)]. As **E**_*j*,*k*_^*σ*^[dSC(*T*, *S*)] denotes performance, each row in a block expresses the dynamics of the performance of a basis technique *T* over another *S*.

In the *S*_2_-RI block, the bottom to top rows of the vertical strip of small squares at the fourth SOSA correspond to subproblem instances 2_4_^2^, 8_4_^2^, 14_4_^2^, 20_4_^2^, and 26_4_^2^ (based from *N*_2_^3.0^ = 〈2,8, 14,20,26〉). The types of changes in the environments that set these subproblem instances are the elements of the vector,

(42) V=〈Ct(2,4),Ct(8,4),Ct(14,4),Ct(20,4),Ct(26,4)〉=〈2,2,2,2,2〉,

where (13) is used to obtain the last line. This result implies that the rows in the vertical strip all correspond to change type 2 which, based on Table 2, denotes an increase in number of tasks. Now, using (16) and (40), we obtain a compact form,

(43) $$\begin{array}{c} V=Ct\left(\langle 2,8,14,20,26\rangle ,4\right)=Ct\left(N_{2}^{3.0},4\right). \end{array}$$

Following a similar approach and using (17), *Nt*(*N*_2_^3.0^, 4) = 〈3,4, 5,6, 7〉. In general, given a vertical strip at SOSA *j* in a block at *S*_*i*_ level, the types of changes and size of increase in the total number of tasks in environments that correspond to the bottom to top rows of this strip can be determined from *Ct*(*N*_*i*_^3.0^, *j*) and *Nt*(*N*_*i*_^3.0^, *j*), respectively. Using (18), *Pt*(*N*_*i*_^3.0^) = 〈*T*_3_, *T*_4_, *T*_5_, *T*_6_, *T*_7_〉 which implies that the bottom to top rows correspond to the subproblems constrained by the task-precedence networks *T*_3_ to *T*_7_, respectively.

Figures 10(b) and 10(c) differ from Figure 10(a) only on their corresponding basis techniques, which are McBA and McBAS, respectively. For example, the fifth small square on the second row of the *S*_3_-McBAR block in Figure 10(b) corresponds to **E**_5,9_^*σ*^[dSC(McBA, McBAR)] where index 9 is the instance label that corresponds to the second row, that is, the second element of *N*_3_^3.0^. Note that, based on Table 5, all of Φ^2^ instances in *N*_1_^3.0^, *N*_2_^3.0^, and *N*_3_^3.0^ correspond to *δ* = 3.0 such that representation format of **E**_*j*,*k*_^*σ*^[·] in these figures is called the *F*_3.0_ format.

The representation format of **E**_*j*,*k*_^*σ*^[·] in Figures 11(a) to 11(c) only differs from that in Figures 10(a) to 10(c) by using

(44) $$\begin{array}{c} N_{1}^{6.0}=\left\{4,10,16,22,28\right\}, \end{array}$$

(45) $$\begin{array}{c} N_{2}^{6.0}=\left\{5,11,17,23,29\right\}, \end{array}$$

(46) $$\begin{array}{c} N_{3}^{6.0}=\left\{6,12,18,24,30\right\} \end{array}$$

instead of *N*_1_^3.0^, *N*_2_^3.0^, and *N*_3_^3.0^, respectively. Note that the rows from the bottom to the top in blocks of Figure 11(a) to 11(c) correspond to Φ^2^ instance labels in *N*_1_^6.0^ to *N*_3_^6.0^, respectively. Based on Table 5, all of Φ^2^ instances in sets *N*_1_^6.0^ to *N*_3_^6.0^ correspond to *δ* = 6.0, such that the representation format of **E**_*j*,*k*_^*σ*^[·] in Figures 11(a) to 11(c) is called the *F*_6.0_ format.

#### 4.5.2. *S*_1_-NDLPOP Block

As mentioned above, the techniques to be analyzed below are NDLPOP, RI, McBA, McBAR, McBAS, and CBAM. Let us now discuss their performances in solving problems set in a dynamic environment. In Figure 10(a), the color of the first square from the left of the bottom row of *S*_1_-NDLPOP block denotes **E**_1,1_^*σ*^[dSC(McBAR, NDLPOP)] > 0. Note that by (13), *Ct*(1,1) = 0 which denotes a type of change in the environment that only involves a change in task duration, based on Table 2. This result implies that McBAR performs better than NDLPOP in solving the first subproblem of instance 1 set in the environment that undergoes only changes in task duration at its first SOSA.

The first to third vertical strips from the left of *S*_1_-NDLPOP block show that **E**_*S*,*Ns*_^*σ*^[dSC(McBAR, NDLPOP)] > 0, ∀*S*, 1 ≤ *S* ≤ 3, and ∀*Ns* ∈ *N*_1_^3.0^ (which corresponds to one vertical strip as explained above). This result implies that McBAR performs better than NDLPOP in solving each of the first to third subproblems of instances in *N*_1_^3.0^. Considering that *Ct*(*Ns*, *S*) = 0, ∀*S*, 1 ≤ *S* ≤ 3, and ∀*Ns* ∈ *N*_1_^3.0^, then the instances are set in environments that undergo changes in task duration at each of their first to third SOSA.

The fourth vertical strip of the *S*_1_-NDLPOP block expresses an abrupt increase in the superiority of performance of McBAR over NDLPOP in solving the fourth subproblems of instances *Ns* ∈ *N*_1_^3.0^. As *Ct*(*Ns*, 4) = 2, based on Table 2, the instances are set in environments that undergo changes in the total number of tasks at their fourth. The fifth to seventh vertical strips of the block show that the superiority of McBAR over NDLPOP generally continues till the seventh subproblems of instances in *N*_1_^3.0^.

However, the first and third rows from the bottom of the eighth vertical strip of the *S*_1_-NDLPOP block show that McBAR is inferior to NDLPOP in solving the eighth subproblems of instances 1 and 13 (which correspond to first and third rows, resp.). As *Ct*(1,8) = 4 and *Ct*(13,8) = 4 (based on Table 2) the instances are set in environments that undergo simultaneous changes in task duration and resource availabilities at their eighth SOSA. The first and third rows from the bottom of the ninth vertical strip of the block show that the inferiority continues to the ninth subproblems of instances 1 and 13 set in environments that undergo changes (types 0 = *Ct*(1,9) and 0 = *Ct*(13,9)) in task duration at their ninth SOSA.

As implied above, McBAR is superior in performance to NDLPOP in solving the first to third subproblems of instances 1 and 13 set in environments that undergo only changes in task duration at each of their first to third SOSA. Thus, McBAR cannot be expected to be inferior to NDLPOP in solving the ninth subproblems of instances 1 and 13 set in the environments that similarly undergo changes in task duration at their ninth SOSA. However, despite the similarity in the type of changes, the inferiority continues from the eighth to the ninth SOSAs of the environments. This suggests inertia in the performance of McBAR.

In contrast, the second, fourth, and fifth rows from the bottom of the eighth and ninth vertical strips of *S*_1_-NDLPOP block express the continuity of superiority in the performance of McBAR against NDLPOP in solving the eighth to the ninth subproblems of instances 7, 19, and 25 (which correspond to the rows, resp.) set in environments that undergo simultaneous changes in resource availability and task duration (at the eighth SOSA) and sole change in task duration (at the ninth SOSA), respectively.

Based on the tenth to the 12th vertical strips of *S*_1_-NDLPOP block, McBAR is generally superior in performance to NDLPOP in solving the tenth to the 12th subproblems of all instances *Ns* ∈ *N*_1_^3.0^. Each of the environments that set the instances undergoes changes (types 5 = *Ct*(*Ns*, 10) and 3 = *Ct*(*Ns*, 11)) that involve an increase in its total number of tasks at its tenth and 11th SOSA and undergoes changes (type 0 = *Ct*(*Ns*, 12)) in task duration at its 12th SOSA.

Referring back to the fourth vertical strip of the *S*_1_-NDLPOP block, the small squares from bottom to top of this strip correspond, respectively, to fourth subproblems of instances in 〈1,7, 13,19,25〉 ( = *N*_1_^3.0^) set in environments that undergo, at their fourth, increases in the total number of tasks by 〈3,4, 5,6, 7〉 ( = *Nt*(*N*_1_^3.0^, 3)), respectively. Based on the colors in the strip and their corresponding increases in the total number of tasks, the superiority in performance of McBAR over NDLPOP in solving the subproblems is not affected by the size of the increase in the total number of tasks at the fourth of the environments that set the instances in *N*_1_^3.0^. Based on the discussions in Section 3.2.1, the subproblems are constrained by task precedence networks illustrated in Figures 6(a), 4, and 6(b) to 6(d), respectively. Thus, the superiority in performance of McBAR to NDLPOP is not affected by the type of network that constrained the subproblems. An analogous conclusion can be drawn from the sixth vertical strip of the block, whose bottom to top rows correspond to 〈2,4, 3,2, 1〉 ( = *Nt*(*N*_1_^3.0^, 6)) increases in the total number of tasks, respectively, and from the tenth vertical strip whose bottom to top rows correspond to 〈2,1, 1,1, 1〉 ( = *Nt*(*N*_1_^3.0^, 10)) increases in the total number of tasks, respectively. Note, however, that the networks are related by being derived from the original network (illustrated in Figure 1) as explained in Section 3.2.1.

#### 4.5.3. *S*_2_-NDLPOP Block

The first and second vertical strips of the *S*_2_-NDLPOP block illustrate that **E**_*S*,*Ns*_^*σ*^[dSC(McBAR, NDLPOP)] > 0, ∀*S*, 1 ≤ *S* ≤ 2, and ∀*Ns* ∈ *N*_2_^3.0^ = 〈2,8, 14,20,26〉 from which *Ct*(*Ns*, *S*) = 0. This implies that McBAR performs better than NDLPOP in solving each of the first and second subproblems of instances in *N*_2_^3.0^ set in environments that undergo changes (type 0 = *Ct*(*Ns*, *S*)) in task duration at each of their first and second SOSAs.

Unlike the case of the *S*_1_-NDLPOP block, the abrupt increase in the superiority in performance of McBAR to NDLPOP is expressed by the third vertical strip of the *S*_2_-NDLPOP block. The bottom to top rows of this strip correspond (as explained in Section 4.5.1) to the instances in *N*_2_^3.0^, respectively, set in the environments that undergo increases in the total number of tasks at their third SOSA.

Based on the rows of the eighth vertical strip of the *S*_2_-NDLPOP block, McBAR is generally inferior in performance to NDLPOP in solving the eighth subproblem of all instances *Ns* ∈ *N*_2_^3.0^. Each of the environments that set the instances undergoes simultaneous changes (type 4 = *Ct*(*Ns*, 8)) in resource availability and duration of tasks at its eighth SOSA. Unlike the case of the *S*_1_-NDLPOP block, based on the ninth vertical strip of *S*_2_-NDLPOP block, this inferiority is not continued to the next (ninth) SOSA of the environments; each undergoes changes (type 6 = *Ct*(*Ns*, 9)) that involve an increase in the total number of tasks. Based on the ninth to 12th vertical strips, McBAR remains superior in performance to NDLPOP at the ninth to 12th SOSAs of the environments.

In the 3rd vertical strip of the *S*_2_-NDLPOP block, the small squares from bottom to top correspond, respectively, to 3rd subproblem of instances in 〈2,8, 14,20,26〉 ( = *N*_2_^3.0^) set in environments that undergo, at their 3rd SOSA, increases in the total number of tasks by 〈3,4, 5,6, 7〉 ( = *Nt*(*N*_2_^3.0^, 6)), respectively. Based on the colors in the strip and their corresponding increases in the total number of tasks, the superiority in performance of McBAR over NDLPOP in solving the subproblems is almost constant with respect to the size of the increase in the total number of tasks. Following the explanation in the last subsection, this superiority is also almost constant with respect to the types of task precedence networks that constrain the subproblems. Analogous conclusions can be drawn from the sixth, ninth, and tenth vertical strips of the *S*_2_-NDLPOP block.

#### 4.5.4. *S*_3_-NDLPOP Block

The first two vertical strips of the *S*_3_-NDLPOP block express the dynamics, of the superiority in performance of McBAR over NDLPOP, similar to that expressed by the first two vertical strips of the *S*_2_-NDLPOP block. The third to fifth rows from the bottom of the fifth vertical strip of the *S*_3_-NDLPOP block illustrate that McBAR is inferior to NDLPOP in solving the fifth subproblems of instances 15, 21, and 27 set in environments that undergo change types 〈4,4, 4〉 = *Ct*(〈15,21,27〉, 5) which are simultaneous changes in task duration and resource availabilities (based on Table 2) at their fifth SOSA. This inferiority continues to be expressed at the third to the fifth rows from the bottom of the sixth and seventh vertical strips of the *S*_3_-NDLPOP block where, correspondingly, the environments undergo change types (〈0,0, 0〉 = *Ct*(〈15,21,27〉, 6) and 〈0,0, 0〉 = *Ct*(〈15,21,27〉, 7)) which are changes in task durations at their sixth and seventh SOSAs, respectively. This result suggests inertia on the performance of McBAR over NDLPOP.

The bottom row of the eighth vertical strip of the *S*_3_-NDLPOP block also shows the inferiority in performance of McBAR over NDLPOP in solving the subproblem of instance 3 set in the environment that undergoes, at its eighth SOSA, simultaneous changes (*Ct*(3,8) = 5) in resource availability and the total number of tasks. The bottom rows of the ninth to 11th vertical strips express the continuity of this inferiority in solving the ninth to 11th subproblems of instance 3. All other unaccounted squares in the *S*_3_-NDLPOP block shows the superiority in performance of McBAR over NDLPOP in solving subproblems that correspond to these squares.

In the third vertical strip of the *S*_3_-NDLPOP block, the small squares from bottom to top correspond, respectively, to the third subproblems of instances in *N*_3_^3.0^ set in environments that undergo, at their third SOSA, increases in the total number of tasks by 〈3,4, 5,6, 7〉 ( = *Nt*(*N*_2_^3.0^, 6)), respectively. Based on the colors in the strip and their corresponding increases in the total number of tasks, the superiority in performance of McBAR over NDLPOP in solving the subproblems is almost constant with respect to the size of the increase in the total number of tasks. Based on the explanation in Section 4.5.2, this superiority is also almost constant with respect to the types of task precedence network that constrain the subproblems. Analogous conclusions can be drawn from the 12th vertical strips of the *S*_3_-NDLPOP block.

#### 4.5.5. Other Blocks

Let us now consider the remaining colored blocks in Figure 10(a). The *S*_1_, *S*_2_, and *S*_3_-RI blocks are generally similar in color profiles to those of the *S*_1_, *S*_2_, and *S*_3_-NDLPOP blocks, respectively. Likewise, the *S*_1_, *S*_2_, and *S*_3_-CBAM blocks are generally similar in color profiles to those of the *S*_1_, *S*_2_, and *S*_3_-NDLPOP blocks, respectively. This implies that the dynamics of McBAR's superiority over RI and CBMA are generally similar to the dynamics of McBAR's superiority over NDLPOP.

The notable exemptions to the last conclusion are as follows. The small squares from the bottom to the top of the first and second vertical strips of the *S*_2_-CBAM, *S*_3_-CBAM, *S*_2_-NDLPOP, *S*_3_-NDLPOP, *S*_2_-RI, and *S*_3_-RI blocks show McBAR as equally, slightly, and highly superior over CBAM, NDLPOP, and RI, respectively. A similar exemption can be drawn on the performances of McBAR over CBAM, NDLPOP, and RI at the small squares from the bottom to the top of the first to the third vertical strips of the *S*_1_-CBAM, *S*_1_-NDLPOP, and *S*_1_-RI blocks, respectively. Following similar reasoning as in Section 4.5.2, the types of changes that correspond to the squares are only on task durations.

The small squares from bottom to top, in the first vertical strip of the *S*_1_-McBA block, show slight superiority in the performance of McBAR over McBA in respectively solving the first subproblems of instances in *N*_1_^3.0^ set in environments that undergo, at their first SOSA, change types 〈0,0, 0,0, 0〉 = *Nt*(*N*_1_^3.0^, 1) which are changes in task duration. Analogous conclusions can be drawn from the second and third vertical strips of the *S*_1_-McBA block.

The fourth to the last vertical strips of the *S*_1_-McBA block generally express the superiority in performance of McBAR over McBA in solving subproblems that correspond to the squares in these strips. Recall from Section 3.5.2 that McBAR differs from McBA in its random immigrant component only. The superiority of McBAR to McBA suggests the effectiveness of this component. Based on their color profile, generally similar performance dynamics are expressed in the *S*_2_ and *S*_3_-McBA blocks. Further, the dynamics of McBAR's performance over McBAS expressed in the *S*_1_, *S*_2_, and *S*_3_-McBAS blocks are generally similar to the dynamics of McBAR's performance over McBA expressed in the *S*_1_, *S*_2_, and *S*_3_-McBA blocks, except that McBAR's performance over McBAS is higher than its performance over McBA.

#### 4.5.6. Other Figures

Let us now discuss performance dynamics, expressed in Figures 10(b) and 10(c), of the NDLPOP, RI, McBA, McBAR, McBAS, and CBAM (included) techniques. Note first that the blank block in Figure 10(b) is under the heading McBA. Thus, as explained in Section 4.5.1, McBA is the basis technique in this figure. The color profiles between blocks under NDLPOP, RI, CBAM, and McBAS headings in Figure 10(a) and blocks under similar headings in Figure 10(b) are, respectively, generally similar which implies that the dynamics of performance, expressed in these blocks, of McBA (the basis technique) over NDLPOP, RI, CBAM, and McBAS are generally similar to those of McBAR. Note that although the dynamics are generally similar, the magnitudes in these dynamics are generally different.

Note that the basis technique in Figure 10(c) is McBAS. The color profiles between blocks under NDLPOP, RI, and CBAM headings in Figure 10(a) and blocks under similar headings in Figure 10(c) are, respectively, generally similar which implies that the dynamics of performance, expressed in these blocks, of McBAS over NDLPOP, RI, and CBAM are generally similar to those of McBAR.

Note that as the differential set coverage dSC(*A*, *B*) in (4) is antisymmetric with respect to its arguments *A* and *B*, so does the average **E**_*S*,*Ns*_^*σ*^[dSC(*A*, *B*)]. For example, **E**_*S*,*Ns*_^*σ*^[dSC(McBAR, McBA)] = −**E**_*S*,*Ns*_^*σ*^[dSC(McBA, McBAR)]. The dynamics of the performance of McBAR over McBA expressed in *S*_1_, *S*_2_, and *S*_3_-McBAR blocks of Figure 10(a) are, respectively, opposite (due to the antisymmetry of **E**_*S*,*Ns*_^*σ*^[·]) to the dynamics of the performance of McBA over McBAR expressed in *S*_1_, *S*_2_, and *S*_3_-McBA blocks of Figure 10(b). A similar relationship holds between McBAR and McBAS.

The dynamics of performances expressed in blocks of Figure 10 are, respectively, generally similar to those expressed in blocks of Figure 11. Considering that instances corresponding to rows in blocks of Figures 10 and 11 correspond, respectively, to *δ* = 3.0 and *δ* = 6.0, as discussed in Section 4.5.1, then the dynamics of the performances of the included techniques are generally similar on the two amounts of *δ*.

## 5. Conclusion and Future Work

In this paper, we investigated multiobjective dynamic resource-constrained project scheduling problems involving an increasing number of tasks. We presented an innovative Evolutionary Algorithm-based approach, called mapping of task ID for centroid-based adaptation with random immigrants (McBAR), and applied this to search for optimal schedules as solutions to the problems. We also presented techniques, other than McBAR, to legitimize subalgorithms of McBAR based on the quality of solutions they obtained in solving the problems.

Compared to the other techniques, McBAR was found to obtain a generally better quality of solutions to the problems. This superiority generally prevails over various types of changes in the environment in which the problems are set. We also supported the legitimacy of several subalgorithms of McBAR through this superiority.

We investigated the dynamics of the performance of McBAR and the other techniques in determining solutions to the problems influenced by various types and sequences of environmental changes. McBAR is found to be generally superior in performance to the other techniques after the first increase in the number of tasks in the environment. Its performance becomes inferior to some of the other techniques when there are simultaneous changes in resources availability and task duration in the environment. This inferiority is generally continued, but diminishing, on few the succeeding environmental changes that do not involve simultaneous changes in resources availability and task duration. This dynamics manifests inertia in McBAR's performance under the influence of environmental dynamics. McBAR's performance does not manifest dependency on the number of new tasks in the environment and on the placement of these new tasks in the task precedence network that constrains the problems. This general stability of McBAR's performances demonstrates the relevance of McBAR for solving the types of dynamical problems investigated in this paper.

In the future, we plan to model McBAR's performance in solving the problems through design of experiments.

## Figures and Tables

**Algorithm 1:**
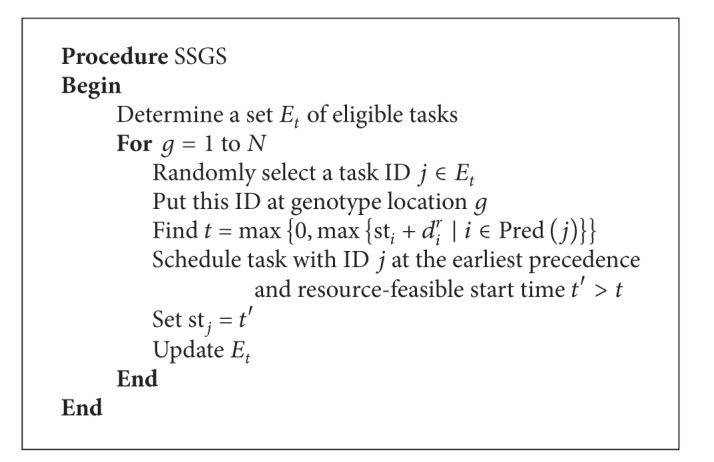
Serial Schedule Generation Scheme (SSGS) Algorithm.

**Algorithm 2:**
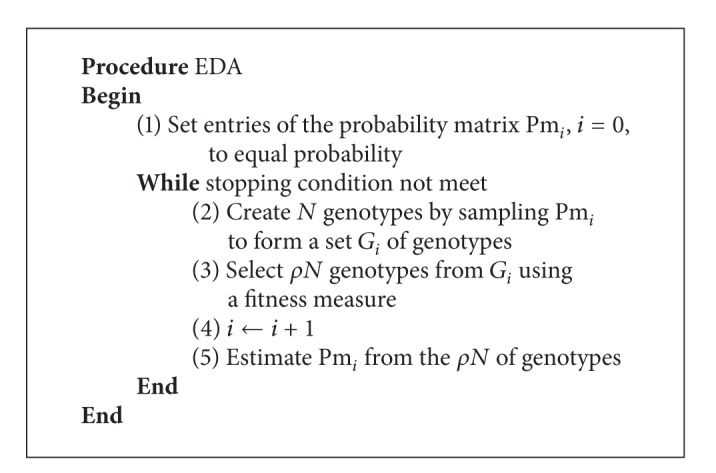
Estimation of Distribution Algorithm.

**Table 1:** Properties of tasks.

Task ID	Duration	*R*_1_ ≤ 16	*R*_2_ ≤ 17	*R*_3_ ≤ 5	*R*_4_ ≤ 16
1	18	4	0	0	0
2	13	10	0	0	0
3	16	0	0	0	3
4	23	3	0	0	0
5	18	0	0	0	8
6	15	4	0	0	0
7	19	0	1	0	0
8	12	6	0	0	0
9	17	0	0	0	1
10	19	0	5	0	0
11	22	0	7	0	0
12	16	4	0	0	0
13	23	0	8	0	0
14	19	3	0	0	0
15	10	0	0	0	5
16	16	0	0	0	8
17	15	0	0	0	7
18	23	0	1	0	0
19	17	0	10	0	0
20	22	0	0	0	6
21	27	2	0	0	0
22	22	3	0	0	0
23	13	0	9	0	0
24	13	4	0	0	0
25	17	0	0	4	0
26	18	0	0	0	7
27	23	0	8	0	0
28	17	0	7	0	0
29	20	0	7	0	0
30	20	0	0	2	0
31	12	6	0	0	0
32	17	0	0	0	1
33	19	0	5	0	0
34	16	0	0	0	8
35	15	0	0	0	7
36	23	0	0	0	0
37	18	0	0	0	7
38	23	0	8	0	0
39	17	0	7	0	0
40	20	0	7	0	0

**Table 2:** Types of environmental changes.

Type	Parameter
0	Task duration
1	Resource availability
2	Total number of tasks
3	Task duration
	Total number of tasks
4	Task duration
	Resource availability
5	Total number of tasks
	Resource availability
6	Task duration
	Total number of tasks
	Resource availability

**Table 3:** Types of sequence of changes.

SOSA	*S*_1_	*S*_2_	*S*_3_	Time
1	0	0	0	4
2	0	0	0	6
3	0	2	6	8
4	2	1	0	12
5	0	0	4	13
6	6	3	0	16
7	1	0	0	19
8	4	4	5	23
9	0	6	1	26
10	5	5	2	30
11	3	0	0	33
12	0	0	3	37

**Table 4:** Types of task number increase sequence (TNIS).

Order	*T*_3_	*T*_4_	*T*_5_	*T*_6_	*T*_7_
1	3	4	5	6	7
2	2	4	3	2	1
3	2	1	1	1	1
4	3	1	1	1	1

**Table 5:** Types of instances.

*δ* = 3.0				*δ* = 6.0		
*S*_1_	*S*_2_	*S*_3_		*S*_1_	*S*_2_	*S*_3_
1	2	3	*T*_3_	4	5	6
7	8	9	*T*_4_	10	11	12
13	14	15	*T*_5_	16	17	18
19	20	21	*T*_6_	22	23	24
25	26	27	*T*_7_	28	29	30

**Table:** (a)

Technique	Population size	Crossover rate	Mutation rate
RI	100	0.9295	0.7
NDLPOP	96	0.8892	0.7
GIBAR	76	0.6813	0.8
CBAM	76	0.6786	0.8
McBA	100	0.7295	0.8
McBAR	76	0.7558	0.7
McBAS	100	0.7282	0.7
MedianBAR	112	0.9775	0.8

**Table:** (b)

Technique	Learning rate	Percentage population
EDA/Φ^2^	0.8	0.8

**Table:** (a)

Techniques	GIBAR	CBAM	NDLPOP	RI	EDA/Φ^2^	McBA	McBAR	McBAS	MedianBAR
GIBAR	N/A	−0.54	0.56	0.37	0.99	−0.64	−0.63	−0.61	−0.65
CBAM	0.54	N/A	0.01	0.23	0.99	−0.35	−0.36	−0.33	−0.38
NDLPOP	−0.56	−0.01	N/A	0.24	1.00	−0.34	−0.36	−0.33	−0.37
RI	−0.37	−0.23	−0.24	N/A	1.00	−0.47	−0.49	−0.45	−0.50
EDA/Φ^2^	−0.99	−0.99	−1.00	−1.00	N/A	−1.00	−1.00	−1.00	−1.00
McBA	0.64	0.35	0.34	0.47	1.00	N/A	−0.07	−0.01	−0.09
McBAR	0.63	0.36	0.36	0.49	1.00	0.07	N/A	0.07	−0.01
McBAS	0.61	0.33	0.33	0.45	1.00	0.01	−0.07	N/A	−0.08
MedianBAR	0.65	0.38	0.37	0.50	1.00	0.09	0.01	0.08	N/A

**Table:** (b)

Technique	GIBAR	CBAM	NDLPOP	RI	EDA/Φ^2^	McBA	McBAR	McBAS	MedianBAR
GIBAR	N/A	−0.58	0.56	0.37	0.99	−0.65	−0.66	−0.62	−0.67
CBAM	0.58	N/A	0.05	0.28	1.00	−0.34	−0.36	−0.31	−0.37
NDLPOP	−0.56	−0.05	N/A	0.25	1.00	−0.37	−0.39	−0.35	−0.38
RI	−0.37	−0.28	−0.25	N/A	1.00	−0.50	−0.51	−0.46	−0.51
EDA/Φ^2^	−0.99	−1.00	−1.00	−1.00	N/A	−1.00	−1.00	−1.00	−1.00
McBA	0.65	0.34	0.37	0.50	1.00	N/A	−0.09	0.00	−0.08
McBAR	0.66	0.36	0.39	0.51	1.00	0.09	N/A	0.11	0.01
McBAS	0.62	0.31	0.35	0.46	1.00	0.00	−0.11	N/A	−0.07
MedianBAR	0.67	0.37	0.38	0.51	1.00	0.08	−0.01	0.07	N/A

**Table 8:** Average execution time.

Technique	*δ* = 3.0	*δ* = 6.0
GIBAR	5.025*E* + 10	5.104*E* + 10
CBAM	5.018*E* + 10	5.082*E* + 10
NDLPOP	4.645*E* + 10	4.714*E* + 10
RI	4.282*E* + 10	4.456*E* + 10
EDA/Φ^2^	4.820*E* + 10	4.921*E* + 10
McBA	5.285*E* + 10	5.330*E* + 10
McBAR	5.428*E* + 10	5.465*E* + 10
McBAS	5.055*E* + 10	5.123*E* + 10
MedianBAR	6.608*E* + 10	6.721*E* + 10

**Table 9:** Average **E**_*t*_^*τ*^[·] performance under change types 0 to 2.

Type	Technique	GIBAR	CBAM	NDLPOP	RI	EDA/Φ^2^	McBA	McBAR	McBAS	MedianBAR
0	GIBAR	N/A	−0.55	0.56	0.34	0.99	−0.59	−0.58	−0.57	−0.61
	CBAM	0.55	N/A	0.03	0.29	0.99	−0.25	−0.23	−0.21	−0.25
	NDLPOP	−0.56	−0.03	N/A	0.29	0.99	−0.25	−0.25	−0.22	−0.25
	RI	−0.34	−0.29	−0.29	N/A	0.99	−0.43	−0.42	−0.38	−0.43
	EDA/Φ^2^	−0.99	−0.99	−0.99	−0.99	N/A	−0.99	−1.00	−1.00	−1.00
	McBA	0.59	0.25	0.25	0.43	0.99	N/A	−0.04	0.01	−0.05
	McBAR	0.58	0.23	0.25	0.42	1.00	0.04	N/A	0.05	−0.02
	McBAS	0.57	0.21	0.22	0.38	1.00	−0.01	−0.05	N/A	−0.07
	MedianBAR	0.61	0.25	0.25	0.43	1.00	0.05	0.02	0.07	N/A

1	GIBAR	N/A	−0.59	0.57	0.40	1.00	−0.73	−0.72	−0.66	−0.74
	CBAM	0.59	N/A	0.05	0.26	1.00	−0.43	−0.48	−0.43	−0.49
	NDLPOP	−0.57	−0.05	N/A	0.23	1.00	−0.44	−0.48	−0.44	−0.50
	RI	−0.40	−0.26	−0.23	N/A	1.00	−0.55	−0.58	−0.53	−0.60
	EDA/Φ^2^	−1.00	−1.00	−1.00	−1.00	N/A	−1.00	−1.00	−1.00	−1.00
	McBA	0.73	0.43	0.44	0.55	1.00	N/A	−0.16	−0.06	−0.15
	McBAR	0.72	0.48	0.48	0.58	1.00	0.16	N/A	0.11	0.04
	McBAS	0.66	0.43	0.44	0.53	1.00	0.06	−0.11	N/A	−0.08
	MedianBAR	0.74	0.49	0.50	0.60	1.00	0.15	−0.04	0.08	N/A

2	GIBAR	N/A	−0.53	0.51	0.19	0.99	−0.82	−0.84	−0.82	−0.84
	CBAM	0.53	N/A	0.03	0.28	0.99	−0.71	−0.73	−0.69	−0.74
	NDLPOP	−0.51	−0.03	N/A	0.24	0.99	−0.74	−0.75	−0.72	−0.75
	RI	−0.19	−0.28	−0.24	N/A	0.99	−0.78	−0.79	−0.77	−0.79
	EDA/Φ^2^	−0.99	−0.99	−0.99	−0.99	N/A	−1.00	−1.00	−1.00	−1.00
	McBA	0.82	0.71	0.74	0.78	1.00	N/A	−0.01	0.02	−0.04
	McBAR	0.84	0.73	0.75	0.79	1.00	0.01	N/A	0.09	−0.07
	McBAS	0.82	0.69	0.72	0.77	1.00	−0.02	−0.09	N/A	−0.08
	MedianBAR	0.84	0.74	0.75	0.79	1.00	0.04	0.07	0.08	N/A

**Table 10:** Average **E**_*t*_^*τ*^[·] performance under change types 3 to 5.

Type	Technique	GIBAR	CBAM	NDLPOP	RI	EDA/Φ^2^	McBA	McBAR	McBAS	MedianBAR
3	GIBAR	N/A	−0.62	0.58	0.14	1.00	−0.90	−0.92	−0.89	−0.92
	CBAM	0.62	N/A	0.07	0.24	1.00	−0.68	−0.76	−0.70	−0.75
	NDLPOP	−0.58	−0.07	N/A	0.16	1.00	−0.71	−0.78	−0.73	−0.76
	RI	−0.14	−0.24	−0.16	N/A	1.00	−0.78	−0.82	−0.78	−0.82
	EDA/Φ^2^	−1.00	−1.00	−1.00	−1.00	N/A	−1.00	−1.00	−1.00	−1.00
	McBA	0.90	0.68	0.71	0.78	1.00	N/A	−0.24	−0.10	−0.20
	McBAR	0.92	0.76	0.78	0.82	1.00	0.24	N/A	0.14	0.05
	McBAS	0.89	0.70	0.73	0.78	1.00	0.10	−0.14	N/A	−0.06
	MedianBAR	0.92	0.75	0.76	0.82	1.00	0.20	−0.05	0.06	N/A

4	GIBAR	N/A	−0.57	0.58	0.40	1.00	−0.21	−0.27	−0.21	−0.26
	CBAM	0.57	N/A	0.02	0.24	1.00	0.16	0.09	0.15	0.09
	NDLPOP	−0.58	−0.02	N/A	0.24	1.00	0.16	0.07	0.11	0.08
	RI	−0.40	−0.24	−0.24	N/A	1.00	0.03	−0.06	0.01	−0.03
	EDA/Φ^2^	−1.00	−1.00	−1.00	−1.00	N/A	−1.00	−1.00	−1.00	−1.00
	McBA	0.21	−0.16	−0.16	−0.03	1.00	N/A	−0.09	0.01	−0.10
	McBAR	0.27	−0.09	−0.07	0.06	1.00	0.09	N/A	0.10	−0.01
	McBAS	0.21	−0.15	−0.11	−0.01	1.00	−0.01	−0.10	N/A	−0.07
	MedianBAR	0.26	−0.09	−0.08	0.03	1.00	0.10	0.01	0.07	N/A

5	GIBAR	N/A	−0.60	0.59	0.09	1.00	−0.68	−0.68	−0.64	−0.68
	CBAM	0.60	N/A	0.03	0.18	1.00	−0.36	−0.42	−0.35	−0.40
	NDLPOP	−0.59	−0.03	N/A	0.15	1.00	−0.39	−0.43	−0.38	−0.42
	RI	−0.09	−0.18	−0.15	N/A	1.00	−0.46	−0.50	−0.45	−0.49
	EDA/Φ^2^	−1.00	−1.00	−1.00	−1.00	N/A	−1.00	−1.00	−1.00	−1.00
	McBA	0.68	0.36	0.39	0.46	1.00	N/A	−0.11	−0.01	−0.10
	McBAR	0.68	0.42	0.43	0.50	1.00	0.11	N/A	0.14	0.02
	McBAS	0.64	0.35	0.38	0.45	1.00	0.01	−0.14	N/A	−0.11
	MedianBAR	0.68	0.40	0.42	0.49	1.00	0.10	−0.02	0.11	N/A

**Table 11:** Average **E**_*t*_^*τ*^[·] performance under change type 6.

Type	Technique	GIBAR	CBAM	NDLPOP	RI	EDA/Φ^2^	McBA	McBAR	McBAS	MedianBAR
6	GIBAR	N/A	−0.54	0.53	0.11	1.00	−0.85	−0.84	−0.81	−0.85
	CBAM	0.54	N/A	0.03	0.19	1.00	−0.63	−0.65	−0.59	−0.64
	NDLPOP	−0.53	−0.03	N/A	0.18	1.00	−0.63	−0.65	−0.60	−0.65
	RI	−0.11	−0.19	−0.18	N/A	1.00	−0.72	−0.73	−0.68	−0.73
	EDA/Φ^2^	−1.00	−1.00	−1.00	−1.00	N/A	−1.00	−1.00	−1.00	−1.00
	McBA	0.85	0.63	0.63	0.72	1.00	N/A	−0.15	−0.02	−0.11
	McBAR	0.84	0.65	0.65	0.73	1.00	0.15	N/A	0.14	0.07
	McBAS	0.81	0.59	0.60	0.68	1.00	0.02	−0.14	N/A	−0.07
	MedianBAR	0.85	0.64	0.65	0.73	1.00	0.11	−0.07	0.07	N/A

## Appendices

### A. Mathematical Formulation of the Static *ϕ*_*i*_^2^ Subproblem

The intuitively described subproblem *ϕ*_*i*_^2^ in Section 3.1 will now formally be defined, a definition mostly copied from [6]. Note that the index *i* in *ϕ*_*i*_^2^ has the range, 0 ≤ *i* ≤ *L* where *L* is given.

#### A.1. Inputs

(a) There are a set *T* of non-pre-emptive tasks
  (A.1) $$\begin{array}{c} T=\left\{T_{0},T_{1},T_{2},\ldots ,T_{N_{t}},T_{N_{t}+1}\right\}, \end{array}$$
where *T*_0_ and *T*_*N*_*t*_+1_ are not tasks but, respectively, central base and ending locations and *N*_*t*_ as the total number of executable tasks. In the foregoing discussion, tasks refer to executable tasks only, except when explicitly stated. Each task *T*_*i*_ will have
  (i) a duration *d*_*i*_ listed in Table 1;
  (ii) a vector **R** = {*R*_*i*_} of required resources by *T*_*i*_: *R*_*i*_ = {*r*_*i*,*j*_} with *i* = 1,…, *N*_*rt*_; *N*_*rt*_ is the number of resource types; *j* = 1,…, *N*_*it*_; *N*_*it*_ is the number of items per type; and *r*_*i*,*j*_ is the *j*th item of resource *R*_*i*_ such as that indicated at the header of Table 1;
(b) a network *N*_*tw*_ of tasks where nodes and arcs represent the tasks and the precedence relations, respectively,
  (A.2) $$\begin{array}{c} N_{tw}=\left(T,A_{rc}\right), \end{array}$$
where *A*_*rc*_ is a set of directed arcs. Pred(*j*) defines a set of direct predecessors, while Succ(*j*) is the set of direct successors of the task *j*. Each task *T*_*i*_ ∈ *T* has a vector *S*_*i*_ = {*s*_*i*,*j*_} where
  (A.3) $$\begin{array}{c} s_{i,j}=\{\begin{array}{cc} 1 & \text{task}\;j\;\text{succeeds}\;i\\ 0 & \text{otherwise}. \end{array} \end{array}$$
(c) A matrix of operational costs *C* = {*c*_*i*,*j*,*k*_}, *i* = 0,…, *N*_*t*_; *j* = 0,…, *N*_*t*_, and *k* = 1,…, *N*_*rt*_. Here *c*_*i*,*j*,*k*_ is the cost of moving resource type *k* from task *i* to task *j*. *c*_*i*,0,*k*_ = 0∀*i*, *k*; that is, no cost is imposed on the return of items to base (the starting point *T*_0_).
(d) The current location of *R*_*i*_'s *j*th items at time *t* is denoted by *l*_*i*,*j*,*t*_^*c*^. Their collection is denoted by **l**_*i*,*t*_^*c*^ = {*l*_*i*,*j*,*t*_^*c*^}, *j* = 0,…, *N*_*it*_. *j* = 0 means that the item is at the central base (*T*_0_).
(e) The previous location of *R*_*i*_'s *j*th items at time *t* is denoted by *l*_*i*,*j*,*t*_^*p*^. Their collection is denoted by **l**_*i*,*t*_^*p*^ = {*l*_*i*,*j*,*t*_^*p*^}. *j* = 0 means that the item was at the central base (*T*_0_).
(f) The state of *R*_*i*_'s *j*th items being moved or not at time *t* is denoted by *m*_*i*,*j*,*t*_^*v*^. Their collection is denoted by **m**_*i*,*t*_^*v*^ = {*m*_*i*,*j*,*t*_^*v*^}:
  (A.4) $$\begin{array}{c} m_{i,j,t}^{v}=\{\begin{array}{cc} 1 & l_{i,j,t}^{c}\neq l_{i,j,t}^{p}\\ 0 & \text{otherwise}. \end{array} \end{array}$$

#### A.2. Constraints

(a) Time constraint: if task *i* is a predecessor of task *j*, then it needs to be completed before starting task *j*,
  (A.5) $$\begin{array}{c} \text{s}{\text{t}}_{i}+d_{i}\leq \text{s}{\text{t}}_{j}, \end{array}$$
  ∀*j*, and ∀*i* ∈ Prec(*j*)
(b) Resource constraint: the amount of being used resources cannot exceed the total amount,
  (A.6) $$\begin{array}{c} r_{i,t}\leq R_{i}, \end{array}$$
  ∀*i* and ∀*t*, where *r*_*i*,*t*_ is the total number of being used items of resource type *R*_*i*_ at time *t*.

#### A.3. Objective Functions

(a) Makespan (*f*_*ms*_) is the time needed to execute an entire schedule, that is, equal to the end time of the last task to be accomplished,
  (A.7) $$\begin{array}{c} f_{ms}=\text{s}{\text{t}}_{l}+d_{l}, \end{array}$$
  where st_*l*_ and *d*_*l*_ are the starting time and the duration of the last task, respectively.
(b) Cost of resource operations (*f*_*cs*_): cost of moving resources between locations of tasks
  (A.8) $$\begin{array}{c} f_{cs}=\underset{t=1\to T_{\max}}{\sum }\;\underset{j=1\to N_{rt}}{\sum }\;\underset{k=1\to N_{it}}{\sum }m_{jkt}^{v}\times c_{l_{jkt}^{p},l_{jkt}^{c},j}. \end{array}$$

#### A.4. Outputs

The products in determining solutions to the *ϕ*_*i*_^2^ subproblem are as follows.

- A vector of start time **s** **t** = {st_*i*_}, with *i* = 1,…, *N*_*t*_ and st_*i*_ as the starting time of task *T*_*i*_.
- The genotype of task IDs is defined in (9).
- The solution to the *ϕ*_*i*_^2^ subproblem is a schedule defined in (10).

### B. Mathematical Descriptions of the Dynamic Φ^2^ Problem

Changes in problem factors, due to environmental dynamics, occur at times denoted by *τ*.

#### B.1. Duration

The dynamic duration of a task *T*_*i*_ is defined as

(B.1) $$\begin{array}{c} d_{i}^{\prime }\left(\tau \right)=N_{m}\left(d_{i},\delta \right)+\delta , \end{array}$$

where *d*_*i*_ is the predefined duration of task with ID *i*; *N*_*m*_ is a normal distribution; and *δ* is the standard deviation. Note that this equation is also used to generate different task duration across simulations of the *ϕ*_*i*_^2^ subproblem. Constraint expression (A.5) is rewritten as follows;

(B.2) $$\begin{array}{c} \text{s}{\text{t}}_{i}+d_{i}^{\prime }\left(\tau \right)\leq \text{s}{\text{t}}_{j}. \end{array}$$

#### B.2. Availability of Resources

We use a function *A*(*τ*)_*v*_ to indicate the availability of resources,

(B.3) $$\begin{array}{c} A_{i,j,\tau }=\mathrm{sign}\left(\tau \right)*l_{i,j,\tau }^{c}, \end{array}$$

where *A*(*τ*)_*v*_ is one, zero, or negative one which correspond to resource being available, broken, or unavailable but at the location |*l*_*i*,*j*,*τ*_^*c*^|.

#### B.3. Number of Tasks

Φ^2^ only has an increase in the total number of tasks. The function representing this increase is

(B.4) $$\begin{array}{c} N_{tw}^{c}=\Gamma_{\text{task}}\left(T^{p},A_{rc}^{p},N_{t}^{p},N_{t}^{c}\right), \end{array}$$

where *T* ^*s*,*p*^, *A*_*rc*_^*p*^, and *N*_*t*_^*p*^ are the sets of tasks and arcs, and the total number of tasks from the previous change, respectively. Further, *N*_*tw*_^*c*^ and *N*_*t*_^*c*^ are the new network and the current total number of tasks, respectively.

#### B.4. Parameters after a Change

Change is applied to the indices of tasks *I* = 〈*I*_1_, *I*_2_,…, *I*_*N*_*t*_^*c*^_〉 after any type of change. However, change is applied to network *N*_*tw*_^*c*^ of (B.4) and the structures of **l**_*i*,*t*_^*c*^, **l**_*i*,*t*_^*p*^, and **m**_*i*,*t*_^*v*^ (defined in items (d) to (f) in Section A.1, resp.) only when there is a change in the total number of tasks.
